# Lacosamide prevents cyclophosphamide-induced testicular dysfunction via inhibition of NF-*κ*B/IL-6/STAT-3 and JNK1/Caspase-3 axes with AR and HO-1 preservation: in vivo and in silico evidence

**DOI:** 10.1007/s00210-026-04994-7

**Published:** 2026-02-02

**Authors:** Mohammed R. A. Ali, Basim A. S. Messiha, Ahmed S. Abdel-Samea, Mina Ezzat Attya, Reham H. Mohyeldin

**Affiliations:** 1 Department of Pharmacology & Toxicology, Faculty of Pharmacy, Beni-Suef University, Beni-Suef, 62514 Egypt; 2 Department of Pharmacology & Toxicology, Faculty of Pharmacy, Deraya University, New Minia City, 61111 Egypt; 3 Department of Pathology, Faculty of Medicine, Minia University, Minia, 61519 Egypt

**Keywords:** Testes, Cyclophosphamide, Lacosamide, JNK1, STAT-3, HO-1

## Abstract

This study investigated lacosamide (LCM), a third-generation antiepileptic drug, as a potential gonadoprotective agent against cyclophosphamide (CP)-induced testicular injury. The research addressed the critical need for safer antiepileptic alternatives in cancer patients requiring concurrent chemotherapy and seizure control. Male Wistar rats underwent comprehensive biochemical and histopathological analyses including Western blot, quantitative reverse transcription polymerase chain reaction (qRT-PCR), immunohistochemistry, steroidogenic enzyme activity assessments, sexual hormone measurements, sperm quality evaluations, and molecular docking studies to evaluate LCM's protective mechanisms against CP-induced testicular damage. CP administration significantly elevated oxidative stress markers, pro-inflammatory mediators (Nuclear factor kappa B [NF-*κ*B], Tumor necrosis factor-α [TNF-α], Interleukin-6 [IL-6], Interleukin-1β [IL-1β]), and apoptotic signaling (Bcl-2-associated x protein [Bax], caspase-3), while activating regulatory proteins c-jun N-terminal kinase 1 (JNK1) and Signal transducer and activator of transcription 3 (STAT-3) (identified as novel markers in CP-induced testicular injury). CP suppressed antioxidant defenses, steroidogenic factors (steroidogenic acute regulatory protein [StAR], 3β-hydroxysteroid dehydrogenase [3β-HSD], 17β-hydroxysteroid dehydrogenase [17β-HSD]), Heme oxygenase 1 (HO-1) expression, and androgen receptor (AR) functionality, resulting in decreased testosterone, gonadotropin levels, and compromised spermatogenesis. LCM treatment effectively mitigated these deleterious effects through multi-target protective mechanisms including c-jun N-terminal kinase 1(JNK1)-mediated apoptosis inhibition, HO-1 upregulation, IL-6/ signal transducer and activator of transcription 3 (STAT-3) signaling suppression, prevention of p-JNK1/STAT-3 crosstalk, and AR preservation. This study establishes LCM's multi-target protective efficacy, supporting its potential as a safer antiepileptic alternative providing dual benefits of seizure control and reproductive preservation during chemotherapy.

**Graphical Abstract:**

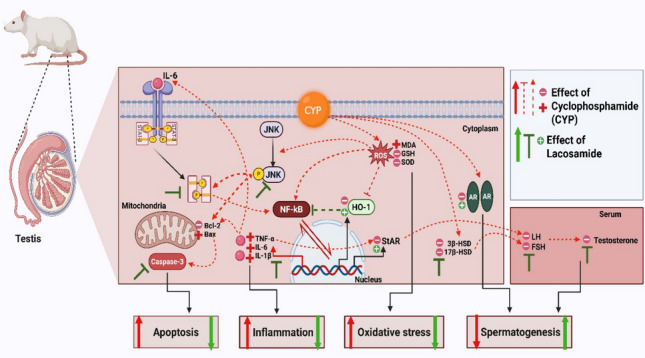

**Supplementary Information:**

The online version contains supplementary material available at 10.1007/s00210-026-04994-7.

## Introduction

Chemotherapeutic interventions significantly compromise male reproductive capacity, with approximately 24% of male recipients experiencing oligospermia or azoospermia (Okada and Fujisawa 2019). Certain chemotherapy medications can decrease sperm production and cause hypogonadism, ultimately manifesting as male subfertility or infertility (Howell and Shalet 2001). This necessitates consideration of fertility preservation strategies before oncological intervention.

Cyclophosphamide (CP) is a potent alkylating chemotherapy agent effective against solid tumors and hematological malignancies by disrupting DNA replication in rapidly dividing cells (Voelcker 2020). Its toxic metabolite acrolein impairs testicular function through multiple pathways: triggering inflammatory responses via Nuclear factor kappa B (NF-*κ*B) and cytokines (Liu et al. 2017). This leads to testicular injury and dysfunction. interleukin-6)IL-6(is a well-established activator of signal transducers and activators of transcription 3 (STAT-3) axis (Wang et al. 2013). Which plays an essential role in several clinical and physiological processes (Hu et al. 2021). It is involved in oxidative stress, and apoptosis. Additionally, CP-induced toxicity is associated with upregulation and phosphorylation of c-Jun N-terminal kinase (JNK), which is a key factor in transmitting cellular signals that regulate genetic expression, cell survival, differentiation, and apoptosis, which can facilitate STAT-3 phosphorylation (Sabapathy et al. 2004; Liu et al. 2012b). Notably, modulation of heme oxygenase-1 (HO-1) expression is a safeguard against various forms of tissue injury, suggesting its potential as a therapeutic target. Investigating CP's impact on steroidogenesis and spermatogenesis through the JNK1/Caspase-3 and NF-*κ*B/IL-6/STAT-3 pathways, alongside HO-1's cytoprotective role, may enable targeted interventions to preserve male fertility during chemotherapy without compromising oncological efficacy.

The therapeutic challenge intensifies when cancer patients require concurrent antiepileptic therapy. First- and second-generation antiepileptic drugs (AEDs) including valproic acid, carbamazepine, phenytoin, levetiracetam, and zonisamide are well-documented to cause testicular dysfunction through oxidative stress and inflammatory mechanisms (Cansu et al. 2011; Osuntokun et al. 2020). This creates a critical therapeutic dilemma where patients requiring both chemotherapy and seizure management face potentially additive reproductive harm.

Lacosamide (LCM), a third-generation antiepileptic drug, demonstrates an exceptional therapeutic profile characterized by efficacy in partial-onset seizures with or without secondary generalization. Its clinical significance is demonstrated as both primary monotherapy and adjunctive treatment (Stöhr et al. 2007; de Biase et al. 2014). LCM is also used to manage epilepsy and neuropathic pain in oncology cases. Several earlier studies have investigated the antioxidant, anti-inflammatory, and anti-apoptotic characteristics of LCM in various animal models, including protection against paclitaxel-induced peripheral neuropathy via suppression of the STAT-3 axis (Al-Massri et al. 2018), lipopolysaccharide (LPS)-induced neuroinflammation (Savran et al. 2019), bilateral cavernous nerve injury-induced erectile dysfunction (Yao et al. 2024), LPS-induced hepatic injury (ÖZmen and İPek 2020), and sepsis-induced urogenital tissue damage in female rats (Gunyeli et al. 2019).

Cancer patients face elevated seizure risk, particularly those with brain tumors (affecting approximately 20% of cancer patients), with epilepsy incidence ranging from 20–80% depending on cancer type and treatment (Weller et al. 2012). CP represents one such chemotherapeutic agent for which seizures have been documented as an adverse effect in oncology patients across multiple case reports and clinical studies (Lin and Chang 2015; Gandhi et al. 2017). While the precise mechanisms remain unclear, this rationale guided our selection of an antiepileptic agent to provide dual benefits: primarily mitigating CP-induced testicular toxicity by targeting JNK1/Caspase-3 and NF-*κ*B/IL-6/STAT-3 pathways and examining HO-1's role, and secondarily preventing potential post-CP seizure activity.

To date, no investigations have examined the potential preventive impact of LCM against CP-induced testicular impairment. While most antiepileptic drugs, particularly first and second-generation agents, induce testicular injury (Al Snafi et al. 2013; Eklioglu and Ilgin 2022), this study represents the first investigation of a third-generation antiepileptic drug as a gonado-protective agent.

## Materials and methods

### Molecular docking

Molecular docking studies were performed to investigate how lacosamide interacts with three specific protein targets: signal transducer and activator of transcription 3 (STAT-3; PDB ID: 6NJS), c-Jun N-terminal kinase 1 (JNK1; PDB ID: 3V3V), and heme oxygenase-1 (HO-1; PDB ID: 3HOK) (Baek et al. 2013; Antar et al. 2022; Jaradat et al. 2023). The crystal structures of these targets were obtained from the RCSB Protein Data Bank and were carefully selected based on their high-resolution quality and the presence of well-defined active sites. Before docking, each protein structure was prepared by removing crystallographic water molecules and any co-crystallized ligands or small molecules using Discovery Studio 2024 Client. Lacosamide was energy-minimized to attain a stable conformation suitable for docking. Docking simulations were performed using AutoDock 4.2. Preparation of the protein targets comprised the addition of polar hydrogen atoms and assignment of partial charges using MGLTools 1.5.6. For each protein, a grid box was generated to determine the docking search space. The grid box for STAT-3 was centered at coordinates (14.561, 55.948, 5.416) with dimensions of 64 × 70 × 66 points. For JNK1, the grid box was centered at (31.500, 45.610, 4.468) with dimensions of 56 × 54 × 64 points. The HO-1 grid box was centered at (22.712, − 1.555, 30.525) and measured 78 × 72 × 64 points. Docking protocol validation was carried out by redocking the co-crystallized ligands into the respective binding sites of each protein, subsequently calculation of the root-mean-square deviation (RMSD) between the docked pose and the crystallographic conformation. The resulting protein–ligand complexes with the lowest binding energy were visualized and analyzed using Discovery Studio 2024 Client to evaluate key binding interactions and conformational orientation within the active site.

### Drugs

Lacosamide (LCM) with a HS Code: (29242990) was obtained from Al Andalous for pharmaceutical industries, Cairo, Egypt. Cyclophosphamide (CP) (Endoxan) with a HS Code: (30049041) was obtained from Baxter Oncology GmbH, Halle, Germany. All additional chemical reagents employed in this experiment were of superior analytical quality and obtained from commercial sources.

### Animals

Fifty mature male Wistar albino rats, aged 8 weeks and weighing 200–240 g were procured from the Animal House of Nahda University in Beni-Suef, Egypt. Prior to commencing the experiment, the rats were provided with one week to adapt to their surroundings. Throughout the experimental period, the animals had ad libitum access to standard pellet food and tap water. The rats were housed under controlled environmental conditions, with a temperature regulated at 25 ± 2 °C and a relative humidity of 45 ± 5%. A 12-h light cycle was also introduced to the rats in a controlled environment (Refaie et al. 2021).

### Experimental design

After sample size calculations (Festing and Altman 2002; Charan and Kantharia 2013), and considering the high mortality rate, rats were randomly divided into five experimental groups (n = 10 per group) as follows:

**Table:** 

Group	Experimental design
Control group	Normal saline was administered intraperitoneally to rats in this group for 7 days
LCM group	Rats were administered LCM at a dosage of 40 mg/kg intraperitoneally for 7 days
CP group	CP was injected on day 6, following 1 h of normal saline injection, the rats were administered a single dosage of CP (200 mg/kg intraperitoneally) to induce testicular toxicity (Salama et al. 2020)
CP + LCM10 group	Rats were administered LCM at a dosage of 10 mg.kg^−1^ intraperitoneally for 7 days (Ahn et al. 2015). On day 6, one hour following the LCM injection, the rats were administered a single dose of CP at a dosage of 200 mg.kg^−1^ intraperitoneally (Salama et al. 2020)
CP + LCM 40 group	Rats were administered LCM at a dose of 40 mg.kg^−1^ intraperitoneally for 7 days (Gunyeli et al. 2019). On day 6, following 1 h of LCM injection, the rats were administered a single dose of CP at 200 mg.kg^−1^ intraperitoneally (Salama et al. 2020)

**Fig. 1:**
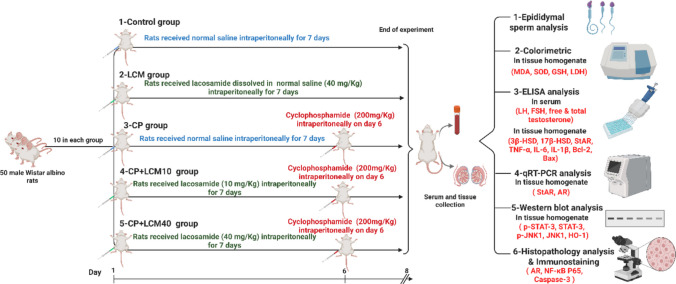
Schematic diagram representing the study workflow to assess lacosamide's testicular protection in cyclophosphamide-treated rats

LCM was prepared fresh daily at a concentration of 10 mg/ml by dissolving LCM powder in normal saline, and CP was prepared at a concentration of 20 mg/ml by dissolving CP powder in normal saline. Appropriate volumes were administered to achieve the desired doses of 10 mg/kg and 40 mg/kg for LCM, and 200 mg/kg for CP. As illustrated in Fig. 1

### Sample collection

The rats were anesthetized on the eighth day of the experiment, utilizing urethane at a dosage of 1.3 g.kg^− 1^; intraperitoneally (Salama et al. 2020) then euthanized by cervical dislocation. Blood samples were obtained by puncturing the heart and then centrifuged at a speed of 4000 xg for 15 min. Aliquots of the serum were obtained and promptly stored at −80 °C to measure reproductive hormone levels (Refaie et al. 2021). The testes and epididymides were dissected. The right testis was rinsed with ice-cold saline (0.9% NaCl) and subsequently bisected into two sections. The first piece was instantly preserved at −80 °C for subsequent qRT-PCR analysis of gene expression. Until the evaluation of the biochemical parameters, the second right testicular segment was kept at a temperature of −80 °C for the ELISA, Western blot, and colorimetric assay, and the parameters were assayed according to manufacturer guidelines and based on established principles described in earlier studies (Noeman et al. 2011; Shah and Maghsoudlou 2016). Concurrently, the left testis was subjected to Bouan's solution for 24 h and prepared for evaluation using histological and immunohistochemical methods (Al-Amoudi 2018). Epididymides have been utilized for the evaluation of sperm quality parameters. As illustrated in Fig. 1.

### Histopathological assessment

Following testis fixation in Bouan’s solution, the tissues were cleaned with 70% ethanol, then the testicular tissues were processed and subsequently incorporated into paraffin wax for producing paraffin blocks (Boran and Ozkan 2004). After that, slices that were Sections of 5 μm in thickness were prepared and placed on slides and stained with hematoxylin and eosin (H&E). A high-quality digital camera was installed on the microscope (U.TV0.5XC-3, Olympus, Tokyo, JP) to observe sections and take photographs. Testicular damage of the seminiferous tubules was scored by Cosentino’s scoring (Cosentino et al. 1985).

**Table:** 

Grade	Characteristics
I	Intact testicular structure
II	The injury had minimal disordered, tightly packed seminiferous tubules and non-cohesive germinal cells
III	There were fewer distinct seminiferous tubule borders and disorganized sloughed germinal cells in the injury
IV	The injury showed seminiferous tubules densely packed with coagulative necrosis of the germinal cells

### Epididymal sperm quality parameters

Fresh cauda epididymides were collected from each group and subsequently minced finely in a normal saline solution. The sperm were expelled into the saline solution via squeezing to assess parameters related to sperm quality, including sperm count, motility, viability, and morphological abnormalities. The hemocytometer slide was employed to count sperm cells under a microscope at a magnification of 400x. Motility was quantified as a percentage of the mobile state (Salama et al. 2020). The eosin-nigrosin stain was employed to evaluate the viability of sperm to differentiate between living and dead sperm (Dibal et al. 2020). Sperm showing morphological abnormalities were detected and recorded (Narayana et al. 2002).

### Determination of reproductive hormone serum levels

Total serum testosterone; (Cat# CSB-E05100r), Free testosterone; (Cat# CSB-E05097r), and Luteinizing hormone (LH); (Cat# CSB-E12654r) ELISA Kits, the kits were procured from Cusabio, TX, USA, and follicle-stimulating hormone (FSH); (Cat# ER0960) ELISA Kit, obtained from Fine Biotech Co, Wuhan, China. All the measurements were conducted while adhering to the instructions provided by the manufacturer (Salama et al. 2020).

### Determination of androgenic enzymes and LDH in tissue homogenate

3β-hydroxysteroid dehydrogenase (3β-HSD); (Cat# RTDL00501), and 17β-hydroxysteroid dehydrogenase (17β-HSD); (Cat# SEF173Ra) ELISA Kits, obtained from Assay Genie, Dublin, Ireland, and CLOUD-CLONE CORP, TX, USA respectively, and lactate dehydrogenase (LDH) that was colorimetrically determined testicular tissue homogenate by using a kit (Cat# 1,001,260) provided by Spinreact, Girona, Spain, the measurements were conducted with adhering to the directives of the manufacturer (Hamza et al. 2023).

### Quantitative real-time polymerase chain reaction for gene expression

Determination of steroidogenic acute regulatory protein (StAR) and androgen receptor (AR) gene expression using quantitative real-time PCR (RT-PCR) (Abdel-Aziz et al. 2024). Total RNA was extracted from testicular tissue using the TRI Reagent (Cat# TR 118) obtained from Molecular Research Center, Ohio, USA, following the directives of the manufacturer. The purity of the RNA was estimated by NanoDrop® spectrophotometer (Thermo Scientific, MA, USA). The GoTaq® 1-Step RT-qPCR System (Cat# A6020) (Promega, WI, USA) was used to ascertain the expression of the target genes and with Maxima SYBR Green Master Mix (Thermo Scientific, MA, USA), following the manufacturer’s instructions. Relative quantification was done using the comparative CT method, normalized to GAPDH, and calculated as follows: RQ = 2ΔΔct (El-Sherbiny et al. 2021). The sequences of the primers were represented in Table 1.

**Table 1:** Primer sequences used for the qRT-PCR

Genes		Primer sequence
*AR*	Forward	5′- TTTGGACAGTACCAGGGACC −3′
	Reverse	5′- CTTCTGTTTCCCTTCCGCAG −3′
*StAR*	Forward	5′- GGGCATACTCAACAACCAG −3′
	Reverse	5′- ACCTCCAGTCGGAACACC −3′
*GAPDH*	Forward	5′- TCACCACCATGGAGAAGGC −3′
	Reverse	5′- GCTAAGCAGTTGGTGGTGCA −3′

### Determination of testicular StAR in tissue

Steroidogenic acute regulatory protein (StAR); (Cat# E2489Ra) was assayed by ELISA Kit, obtained from BT LAB, Shanghai, China, the measurements were conducted under the manufacturer's guidelines (Hamza et al. 2023).

### Determination of oxidative stress markers in tissue

Testicular malondialdehyde (MDA); (Cat# MD 25 29), superoxide dismutase (SOD); (Cat# SD 25 21), and reduced glutathione (GSH); (Cat# GR 25 11), were all determined colorimetrically in the supernatant of testicular tissue homogenate pursuant to the directives of the manufacturer. All kits were acquired from Biodiagnostic, Giza, Egypt (Abu-Risha et al. 2022).

### Determination of inflammatory markers in testicular tissue

Testicular tumor Necrosis Factor Alpha (TNF-α); (Cat# E-EL-R0019), interleukin 6 (IL-6); (Cat# E-EL-R0015), and interleukin 1β (IL-1β); (Cat# E-EL-R0012), were assessed by utilizing ELISA, following the instructions provided by the manufacturer, Elabscience, TX, USA (Abu-Risha et al. 2022).

### Determination of Bcl-2 and Bax in tissue

B-cell lymphoma 2 (Bcl-2); (Cat# E-EL-R0096), and Bcl-2 associated x protein (Bax); (Cat# E-EL-R0098) were assayed using ELISA kits, according to the given instructions by the manufacturer Elabscience, TX, USA.

### Immunohistochemical assessment

To carry out the immunostaining technique, deparaffinized testicular sections were put onto coated slides, in compliance with the specifications provided by the manufacturer. The primary antibodies were applied to the slides overnight at 4°C. For the androgen receptor (AR) Rabbit pAb; (Cat# A16200) was obtained from abclonal, MA, USA, NF-*κ*B P65 Rabbit pAb; (Cat# bs-20159R) from Bioss, MA, USA, and caspase-3 Rabbit pAb; (Cat# A11953) was obtained from abclonal, MA, USA (Abu-Risha et al. 2022). The samples were incubated with an HRP-conjugated secondary antibody, and the signal was detected using a peroxide substrate with 3,3′-diaminobenzidine (DAB) as the chromogen. A high-quality digital camera was utilized to visualize specimens and capture photographs, which were then connected to an Olympus light microscope (U.TV0.5XC-3). The quantity of AR, NF-*κ*B P65, and caspase-3 was calculated from the average of the % area expression using Image J Software.

### Western blotting analyses for protein expression

The testes were homogenized in ice-cold radio-immunoprecipitation assay (RIPA) buffer with the addition of 1% protease inhibitor and phosphatase inhibitor cocktail (Santa Cruz Biotechnology; Cat# sc-29130) and phosphatase inhibitor cocktail (Santa Cruz Biotechnology; Cat# sc-45044). Afterwards, the samples' protein content was assessed using spectrophotometry using the Bicinchoninic acid assay method (Santa Cruz Biotechnology; Cat# sc-278767A). Following quantification, samples were denatured by boiling in 4 × Laemmli buffer. Equal protein amounts (typically 20–50 μg) were separated by SDS-PAGE (8%, 10%, or 14% gels) based on the molecular weight of target proteins. Proteins were then transferred onto Polyvinylidene fluoride or polyvinylidene difluoride (PVDF) using Mini Trans-Blot® Cell (BioRad, Hercules, CA, USA). PVDF membranes were blocked with 5% bovine serum albumin (BSA) in TBST (1 × TBS, 0.1% Tween-20) for 1 h at room temperature, then probed with primary antibodies diluted in 3% BSA/TBST overnight at 4 °C as blocking buffer and antibody diluent with signal transducer and activator of transcription 3 (STAT-3) and c-Jun-N-terminal protein kinase1 (JNK1) as we measured its total and phosphorylated forms which interfere with usage of non-fat dry milk. Heme Oxygenase 1 (HO-1), so we alternatively used 5% non-fat skim dry milk as a blocking and diluent buffer since there is no phosphorylation interference with it. Primary mouse monoclonal antibodies against STAT-3; (1:500; Cat# sc-8019), and phospho-STAT-3 (Tyr705; 1:300; Cat# sc-8059), JNK1; (1:200; Cat# sc-1648), phospho-JNK1 (Thr183/Tyr185; 1:200; Cat# sc-6254), HO-1; (1:300; Cat# sc-390991), and β-Actin Antibody (1:1000; Cat# sc-47778) were purchased from Santa Cruz Biotechnology (Dallas, TX, USA). After 4 washes in 1 × TBST, the blots were incubated with KPL alkaline phosphatase (AP)-conjugated goat anti-mouse (Cat# 5220–0310), (1:10,000), secondary antibodies (SeraCare, Milford, MA, USA), and the protein bands were visualized by incubation with 1-Step™ nitro blue tetrazolium/5-bromo-4-chloro-3-indolyl-phosphate (NBT/BCIP) substrate solution (Thermo Fisher Scientific, Waltham, MA, USA). Protein expression levels were normalized to β-actin, then normalized to the control group, we calculated the ratios of normalized phosphorylated forms to normalized total forms and band intensities were quantified using Image J Software (Abdel-Aziz et al. 2024; Zhang et al. 2024).

### Statistical analysis

The data are presented as mean ± standard deviation. The software applied was GraphPad Prism 7 (GraphPad Software, CA, USA) to do statistical analysis. The significance of the group means was determined using one-way ANOVA for normally distributed data, and the mean values were compared pairwise using Tukey's post hoc test. The histopathological data are expressed as median (Interquartile Range (IQR)), were statistically compared between groups by the non-parametric Kruskal–Wallis test followed by the Mann–Whitney U-test for pairwise comparisons. Pearson correlation analysis was performed to assess relationships between measured parameters using group means. Group comparison and correlation heatmaps were generated using GraphPad Prism to visualize treatment effects and parameter relationships. If the p-value was less than 0.05, it was considered a significant difference. To determine whether the data followed a normal distribution, the Shapiro–Wilk test was applied. Sample size power was calculated using GPower software to ensure that sample size achieved an accepted power (> 0.8) with the least type II error. After calculations, our sample size statistical power was greater than 0.95.

## Results

### Molecular docking of LCM with STAT-3, JNK1, and HO-1 proteins

**Fig. 2:**
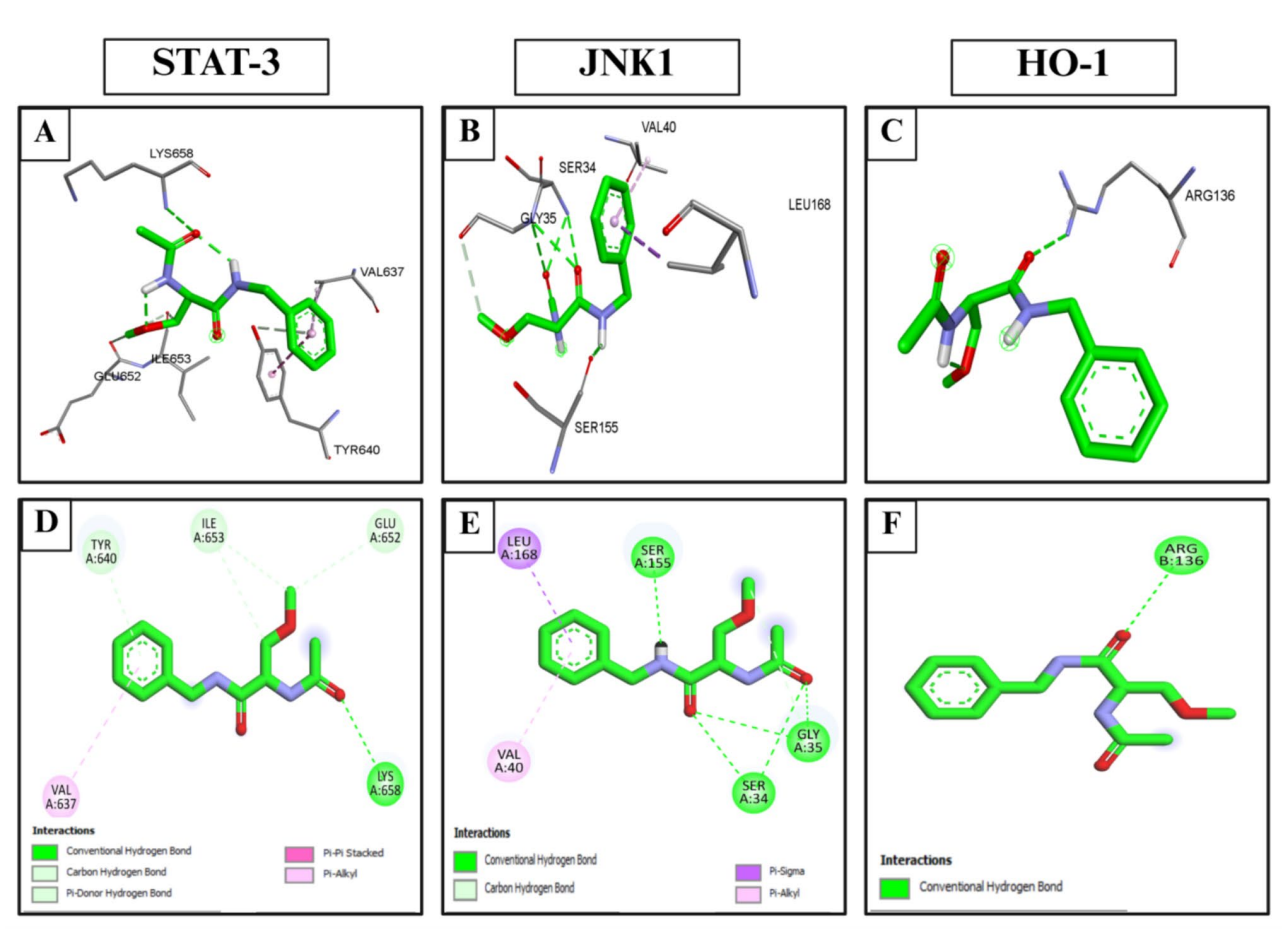
The molecular docking results: (**A**), (**B**), (**C**) 3D binding mode of lacosamide with STAT-3, JNK1, and HO-1, respectively. (**D**), (**E**), (**F**) 2D binding mode of LCM with STAT-3, JNK1, and HO-1, respectively. Heme oxygenase 1, HO-1; JNK1, c-Jun N-terminal kinase 1; Lacosamide, LCM; STAT-3, Signal transducer and activator of transcription 3

Molecular docking was performed to evaluate that LCM is bound to STAT-3, JNK1, and HO-1. The observed RMSD values were 1.39 Å for STAT-3, 0.395 Å for JNK1, and 1.80 Å for HO-1, all of which fall within acceptable limits for validating docking reliability. LCM exhibited a strong binding affinity to these targets. The binding affinity of LCM to key targets is shown in Table 2. The binding interactions between LCM and each target are shown in (Fig. 2A-F). The results showed that LCM has a strongly binding affinity to HO-1 comparable to the co-crystallized ligand, followed by binding affinity with STAT-3 and JNK1. Carbonyl groups of LCM have a key role in binding to the selected targets.

**Table 2:** The affinity of lacosamide and the co-crystallized ligand to key targets

Targets	Affinity of LCM (kcal/mol)	Affinity of co-crystallized ligand (kcal/mol)
STAT-3	−4.84	−8.59
JNK1	−5.19	−9.29
HO-1	−6.26	−8.5

Heme oxygenase 1, HO-1; JNK1, c-Jun N-terminal kinase 1; Lacosamide, LCM; STAT-3, Signal transducer and activator of transcription 3

### Histopathological results

**Fig. 3:**
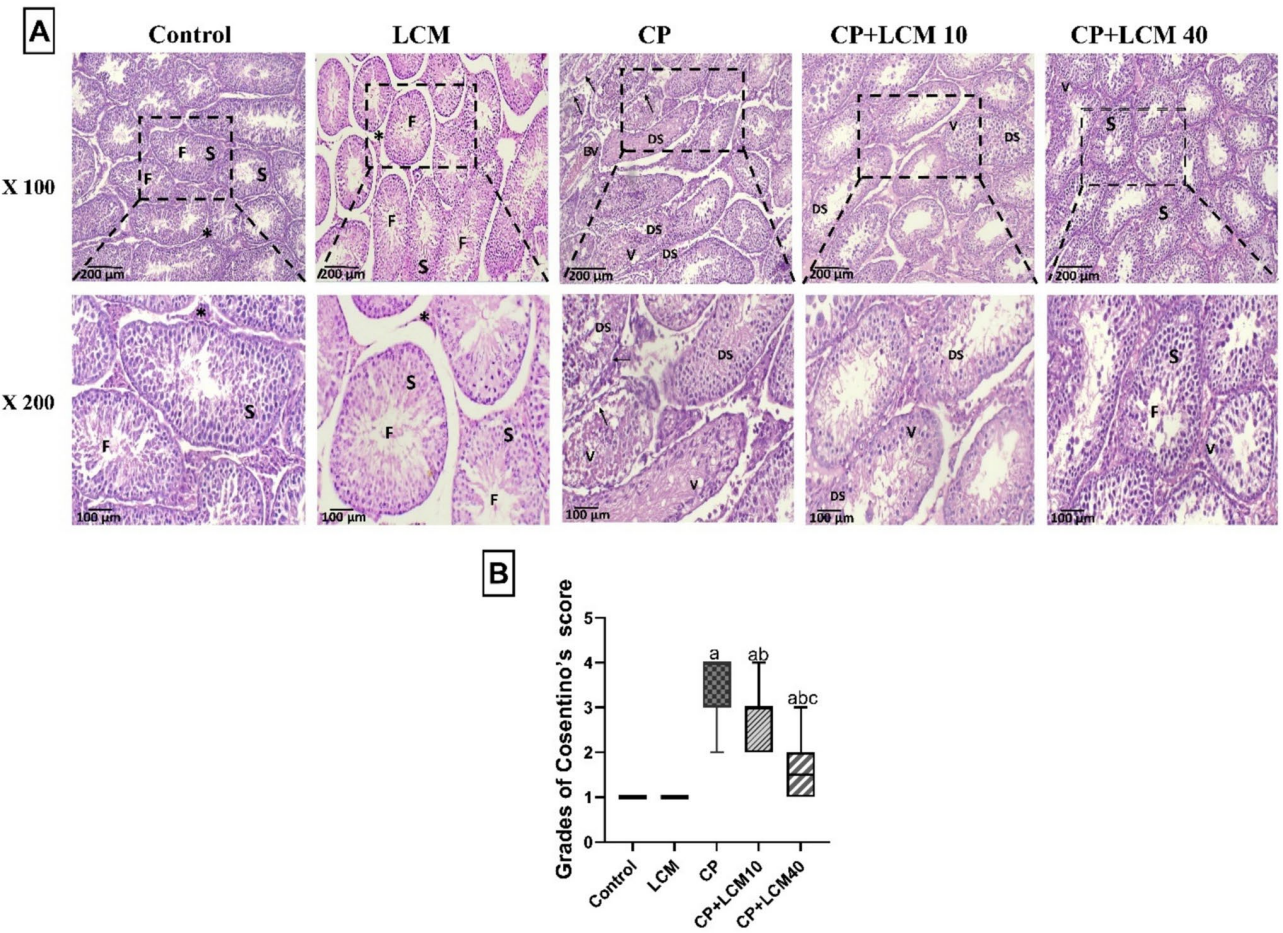
Histopathological testicular sections. (**A**) H & E staining (X 100, scale bar = 200 μm, X 200, scale bar = 100 μm); control group; LCM group; demonstrated normal architecture of the seminiferous tubules and spermatogenic cells (S), free sperms filling the lumen (F), and normal Leydig cells (*). CP group showed testicular degeneration accompanied by vacuolation (V), reduction in the thickness of the germinal epithelium, irregular borders of some seminiferous tubules (arrows) with exfoliated and degenerated spermatogenic cells (DS), and observed congested and dilated blood vessels (BV). The CP + LCM10 group showed some vacuolated cells (V) and fewer seminiferous tubules with exfoliated and degenerated spermatogenic cells (DS) than the CP group. CP + LCM40 group showed mild alteration in the structure of seminiferous tubules with few vacuolated cells (V), and significant increase in regular structure of the seminiferous tubules (S) with free sperm within the seminiferous tubules lumen (F); (**B**) Cosentino’s score of histopathological examination. Data are expressed using a box and whisker plot with median (IQR), (*n* = *10*). In comparison to the control group, the CP group, and the CP + LCM10 group, the corresponding *p*-values are less than 0.05 (^a^, ^b^, and ^c^, respectively). CP, cyclophosphamide; LCM, lacosamide

Sections of the testis from both the LCM and normal control groups showed the usual spermatogenic cell and seminiferous tubule architecture. CP showed a significantly worse histopathological score, exhibiting a 72.2% increase in histopathological alterations compared to control rats, and showed testicular degeneration, characterized by vacuolation and necrosis. The germinal epithelium becomes thinner, and certain seminiferous tubules with exfoliated and degenerated spermatogenic cells have irregular borders, In this group, approximately 70% of rats exhibited score IV, 20% demonstrated score III, and 10% presented with score II. The CP + LCM10 group showed significantly lower scores, resulting in a 25% improvement relative to the CP rats with vacuolated cells and inconsistent borders of some seminiferous tubules, along with a greater presence of exfoliated and degraded spermatogenic cells in comparison to the CP group, In this group, approximately 10% of rats exhibited score IV, 50% demonstrated score III, and 40% presented with score II. In addition, the CP + LCM40 group demonstrated a more substantial protective effect, with a 55.6% improvement in comparison to the compared to CP group and showed preserved architecture of tubules with ordered spermatogenic cells, some exhibiting sperm flagella, while other regions are distinct, score distribution in this group was: 10% score III, 40% score II, and 50% score I (Fig. 3A–B).

### Effect of LCM on epididymal sperm quality parameters

As shown in Table 3, CP-treated rats exhibited substantial alteration in sperm parameters as compared to control rats: 45.4% reduction in sperm count (*p* < 0.0001), 46.4% reduction in sperm motility (*p* < 0.0001), 46.7% reduction in sperm viability (*p* < 0.0001), accompanied by an 81.88% rise in sperm abnormalities (*p* < 0.0001) from the control group. The characteristics exhibited substantial enhancement in the CP + LCM10 and CP + LCM40 groups relative to the CP-treated group, specifically: increases of 25.5% and 38.65% in sperm count (*p* < 0.0001 for both), 36.22% and 39.57% in sperm motility (*p* < 0.0001 for both), 33.64% and 41.70% in sperm viability (*p* < 0.0001 for both), and decreases of 49.7% and 70% in abnormal sperm (*p* < 0.0001 for both).

**Table 3:** Effect of LCM on epididymal sperm quality parameters (sperm count, sperm mobility, sperm viability, and sperm abnormality)

Groups	Parameters			
	Sperm count (million/ml)	Sperm mobility (%)	Sperm viability (%)	Sperm abnormality (%)
Control	98.37 ± 7.277	83.24 ± 4.365	85.18 ± 4.170	10.69 ± 2.041
LCM	96.66 ± 5.561	83.17 ± 4.090	84.27 ± 4.077	10.78 ± 2.288
CP	53.73 ± 5.370^a^	44.65 ± 5.203^a^	45.36 ± 4.355^a^	59.00 ± 6.381^a^
CP + LCM 10	72.12 ± 6.521^ab^	69.99 ± 6.099^ab^	68.35 ± 5.093^ab^	29.65 ± 5.316^ab^
CP + LCM 40	87.57 ± 5.183^abc^	73.89 ± 6.029^ab^	77.80 ± 4.680^abc^	17.72 ± 3.647^abc^

The data is shown as a mean ± standard deviation, (*n* = *10*). When compared to the control group, the CP group, and the CP + LCM10 group, the corresponding *p*-values are less than 0.05 (^a^, ^b^, and ^c^, respectively). CP, cyclophosphamide; LCM, lacosamide

### Effect of LCM on reproductive hormone serum levels

**Fig. 4:**
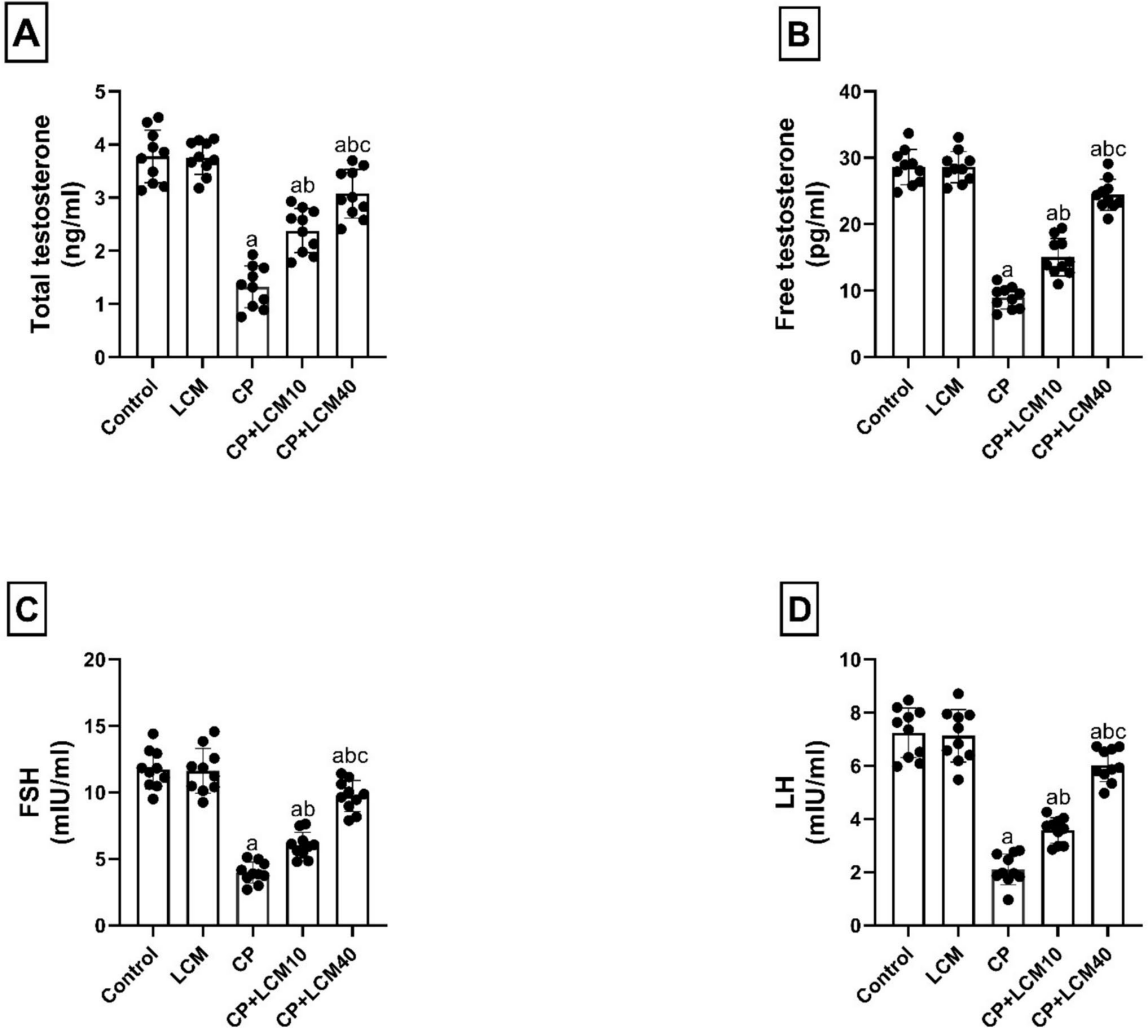
Effect of LCM on reproductive hormone levels in serum in experimental groups. (**A**) total testosterone; (**B**) free testosterone; (**C**) FSH; (**D**) LH. The bars demonstrate the mean ± SD, (*n* = *10*); In comparison to the control group, the CP group, and the CP + LCM10 group, the corresponding *p*-values are less than 0.05 (^a^, ^b^, and ^c^, respectively). CP, cyclophosphamide; FSH, follicle-stimulating hormone; LCM, lacosamide; LH, luteinizing hormone

CP treatment significantly decreased serum sex hormones versus controls (total testosterone: 64.9%, *p* < 0.0001; free testosterone: 68.8%, *p* < 0.0001; FSH: 66.2%,* p* < 0.0001; LH: 70.9%, *p* < 0.0001). Both LCM doses (10 and 40 mg/kg) effectively reversed these reductions as follows: 44.41% and 56.95% increases in total testosterone (*p* < 0.0001 for both), 40.68% and 63.45% increases in free testosterone (*p* < 0.0001 for both), 34.29% and 59.26% increases in FSH (*p* = 0.0051 and *p* < 0.0001), and 41.05% and 65.06% increases in LH (*p* = 0.0006 and *p* < 0.0001) (Fig. 4A-D).

### Effect of LCM on androgenic enzymes and LDH in testicular tissue

**Fig. 5:**
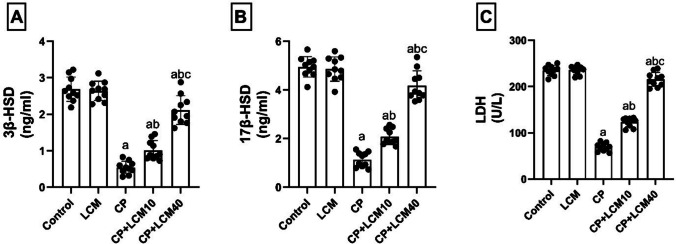
Effect of LCM on androgenic enzymes and LDH in testicular tissue in experimental groups. (**A**) 3β-HSD; (**B**) 17β-HSD; (**C**) LDH. The bars demonstrate the mean ± SD, (*n* = *10*); In comparison to the control group, the CP group, and the CP + LCM10 group, the corresponding *p*-values are less than 0.05 (^a^, ^b^, and ^c^, respectively). CP, cyclophosphamide; LDH, lactate dehydrogenase; LCM, lacosamide; 3β-HSD, 3β-hydroxysteroid dehydrogenase; 17β-HSD, 17β-hydroxysteroid dehydrogenase

According to (Fig. 5A-C), the levels of 3β-HSD, 17β-HSD, and LDH proteins were markedly reduced in CP-treated rats by 79.9% (*p* < 0.0001), 77.1% (*p* < 0.0001), and 70.7% (*p* < 0.0001), respectively, in relation to those in control rats. Inversely, these levels were elevated in CP + LCM10 and CP + LCM40 groups as follows: 46.8% and 74.45% increase in 3β-HSD (*p* = 0.007 and *p* < 0.0001), 45.3% and 72.83% increase in 17β-HSD (*p* = 0.0002 and *p* < 0.0001), and 43.91% and 67.96% increase in LDH (*p* < 0.0001 for both), respectively, when compared to rats in CP group.

### Effect of LCM on StAR and AR in testicular tissue

**Fig. 6:**
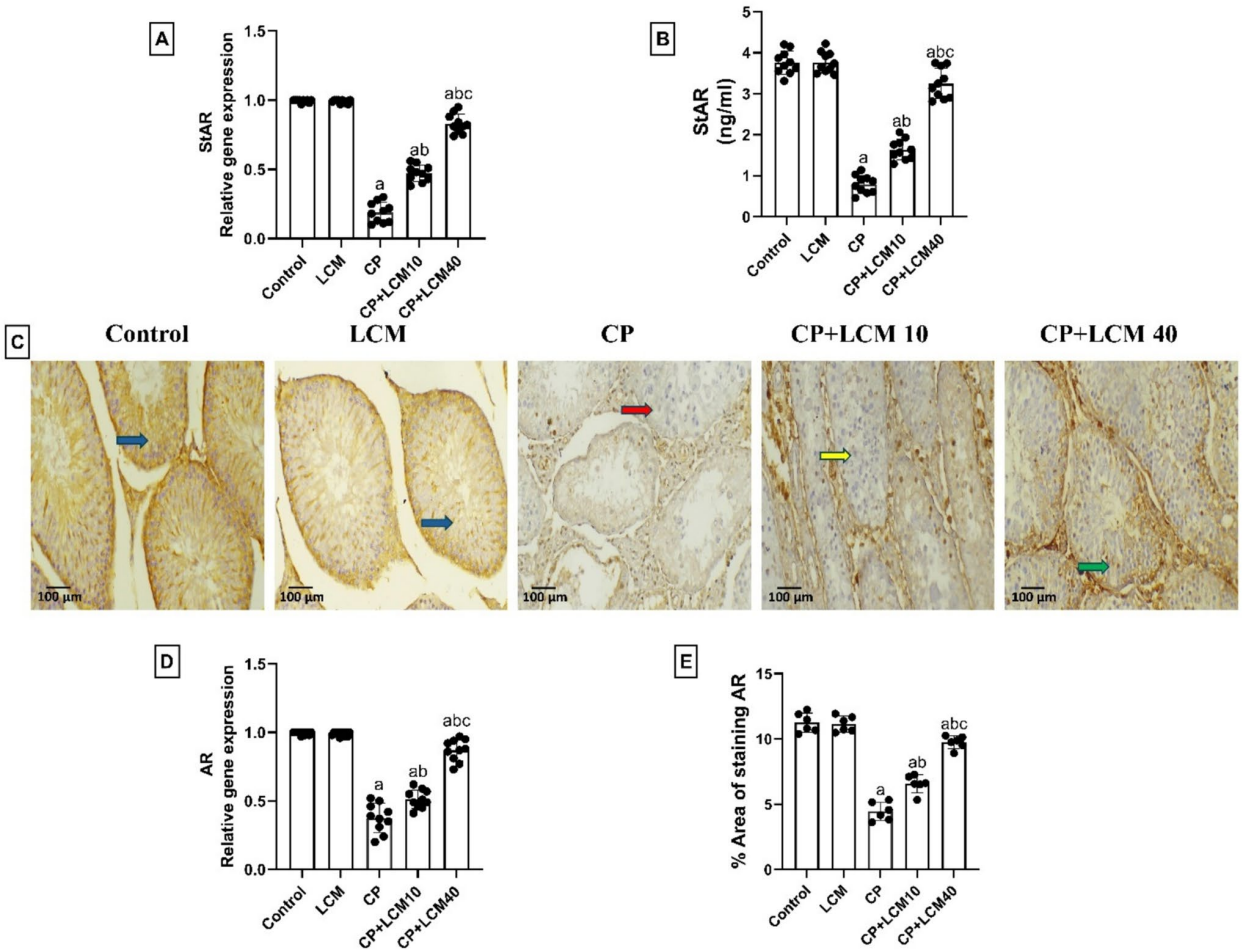
Effect of LCM on StAR and AR in testicular tissue in experimental groups. (**A**) Relative mRNA expression of StAR; (**B**) Testicular StAR level; (**C**) Representative testicular AR immunohistochemical staining photomicrographs indicate expression of AR. Positive staining was brownish yellow. Strong expression is observed in control and LCM groups (blue arrows), with marked downregulation in CP group (red arrows). CP + LCM10 group shows intermediate expression (yellow arrows), while CP + LCM40 group demonstrates substantial upregulation (green arrows). (X 200, scale bar = 100 μm); (**D**) Relative mRNA expression of AR. (**E**) Area percentage of AR immunohistochemical expression. The Data are expressed using bar plot with the mean ± SD, (*n* = *10* for mRNA expression of StAR and AR, StAR ELISA assays, and *n* = *6* for AR immunohistochemical assay); In comparison to the control group, the CP group, and the CP + LCM10 group, the corresponding *p*-values are less than 0.05 (^a^, ^b^, and ^c^, respectively). AR, androgen receptor; CP, cyclophosphamide; LCM, lacosamide; StAR, steroidogenic acute regulatory protein

As shown in (Fig. 6A-B), the comparable data from qRT-PCR analysis showed that CP-exposed rats had significant downregulation of StAR mRNA expression in the testis by 81% (*p* < 0.0001), and after the cotreatment with LCM, upregulation of the gene expression was elevated in CP + LCM10 and CP + LCM40 groups as follows: 59.96% and 77.23% increase (*p* < 0.0001 for both), respectively. To further validate these gene data, ELISA techniques for StAR measurement were performed. As a result, CP-exposed rats had a significant downregulation of StAR in the testis by 78.9% (*p* < 0.0001), in relation to those in control rats. Inversely, after the cotreatment with LCM, upregulation in CP + LCM10 and CP + LCM40 groups by 51.62% and 75.58% (*p* < 0.0001 for both), respectively.

AR mRNA expression assessed by qRT-PCR analysis showed that rats of the CP group had significant downregulation of AR mRNA expression in the testis by 62.1% (*p* < 0.0001), and after the administration of LCM, the gene expression was elevated in CP + LCM10 and CP + LCM40 groups as follows: 26.85% and 56.78% increase (*p* = 0.0003 and *p* < 0.0001), respectively when compared to rats in CP group. To further validate these gene data, immunohistochemical staining of AR was applied. As a result, CP-exposed rats had a considerable decrease in the intensity of AR staining in the testes (*p* < 0.0001) in relation to that in control rats. Inversely, after the cotreatment with LCM, AR staining in CP + LCM10 and CP + LCM40 groups showed a marked higher intensity from the CP group in a dose-dependent manner (*p* < 0.0001 for both) (Fig. 6C-D).

### Effect of LCM on oxidative stress markers in testicular tissue

**Fig. 7:**
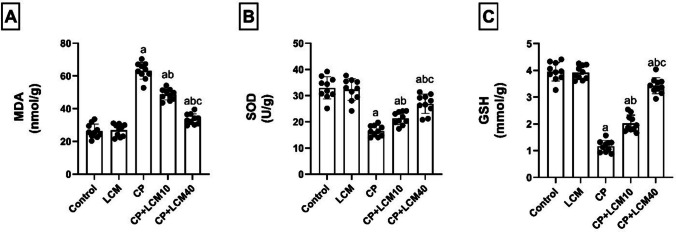
Effect of LCM on levels of oxidative stress markers in testicular tissue in experimental groups. (**A**) MDA; (**B**) SOD; (**C**) GSH. The bars demonstrate the mean ± SD, (*n* = *10*); In comparison to the control group, the CP group, and the CP + LCM10 group, the corresponding *p*-values are less than 0.05 (^a^, ^b^, and ^c^, respectively). CP, cyclophosphamide; GSH, reduced glutathione; LCM, lacosamide; MDA, malondialdehyde; SOD, superoxide dismutase

CP exhibited disruption of the oxidant/antioxidant balance, shown by a noticeable rise in the concentration of MDA in the testes by 58.17% (*p* < 0.0001) from the control rats. This level was decreased in rats of CP + LCM10 and CP + LCM40 groups by 22.8% and 46.6% (*p* < 0.0001 for both), respectively, as compared with the CP group. Inversely, CP-treated rats had substantially lower levels of the antioxidants SOD (by 49.9%, *p* < 0.0001) and GSH (by 70.5%, *p* < 0.0001) compared to control rats. The antioxidant defense capacity was significantly elevated in CP + LCM10 and CP + LCM40 rats compared to CP group, as demonstrated by 22.61% and 38.52% in SOD (*p* = 0.022 and *p* < 0.0001) and 42.29% and 65.99% in GSH (*p* < 0.0001 for both), respectively (Fig. 7A–C).

### Effect of LCM on the inflammatory cascade in testicular tissue

**Fig. 8:**
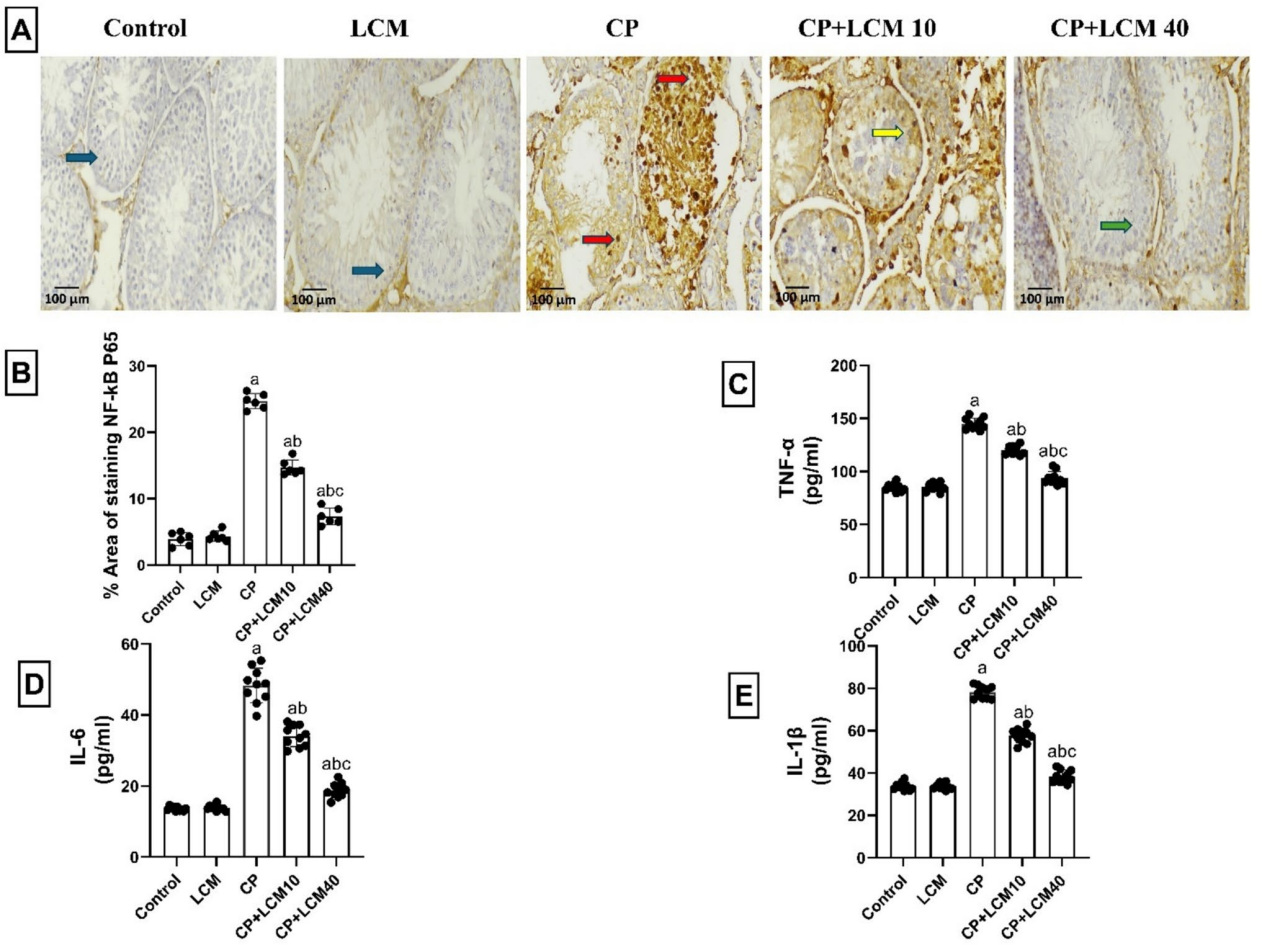
Effect of LCM on levels of inflammatory markers in testicular tissue in experimental groups. (**A**) Representative testicular nuclear factor-*κ*B P65 (NF-*κ*B P65) immunohistochemical staining photomicrographs indicate expression of NF-*κ*B P65. Positive staining was brownish yellow. Minimal expression is observed in control and LCM groups (blue arrows), with marked upregulation in CP group (red arrows). CP + LCM10 group shows intermediate expression (yellow arrows), while CP + LCM40 group demonstrates substantial downregulation (green arrows). (X 200, scale bar = 100 μm); (**B**) Area percentage of NF-*κ*B P65 immunohistochemical expression; (**C**) TNF-α; (**D**) IL-6; (**E**) IL-1β. The Data are expressed using bar plot with the mean ± SD, (*n* = *6* for NF-*κ*B P65 immunohistochemical assay and *n* = *10* for TNF-α, IL-1β, and IL-6 assays); In comparison to the control group, the CP group, and the CP + LCM10 group, the corresponding *p*-values are less than 0.05 (^a^, ^b^, and ^c^, respectively). CP, cyclophosphamide; IL-6, Interleukin 6; IL-1β, Interleukin 1β; LCM, lacosamide; TNF-α, tumor necrosis factor-alpha

Inflammation-related factors were determined in the testicular tissue. NF-*κ*B was shown to be highly expressed, and the stain was intense in testicular sections from rats that were treated with CP (*p* < 0.0001), according to immunohistochemical imaging, and inversely, the low dose of LCM (10mg/kg) showed lower intensity than the CP group (*p* < 0.0001). In addition, the LCM (40mg/kg) lowered NF-*κ*B expression significantly than the CP and CP + LCM10 groups (*p* < 0.0001). The increase in NF-*κ*B expression was associated with considerably elevated levels of pro-inflammatory mediators; TNF-α, IL-6, and IL-1β by 41.37% (*p* < 0.0001), 71.86% (*p* < 0.0001), and 56.84% (*p* < 0.0001), respectively, were observed in the testicular tissue of CP rats when in comparison to control animals. Conversely, these levels were decreased in CP + LCM10 and CP + LCM40 groups as follows: 17.2% and 35.3% decrease in TNF-α (*p* < 0.0001 for both), 29.5% and 61.2% decrease in IL-6 (*p* < 0.0001 for both), and 26.3% and 50.8% decrease in IL-1β (*p* < 0.0001 for both), respectively, when compared with the CP group (Fig. 8A–E).

### Effect of LCM on apoptotic markers in testicular tissue

**Fig. 9:**
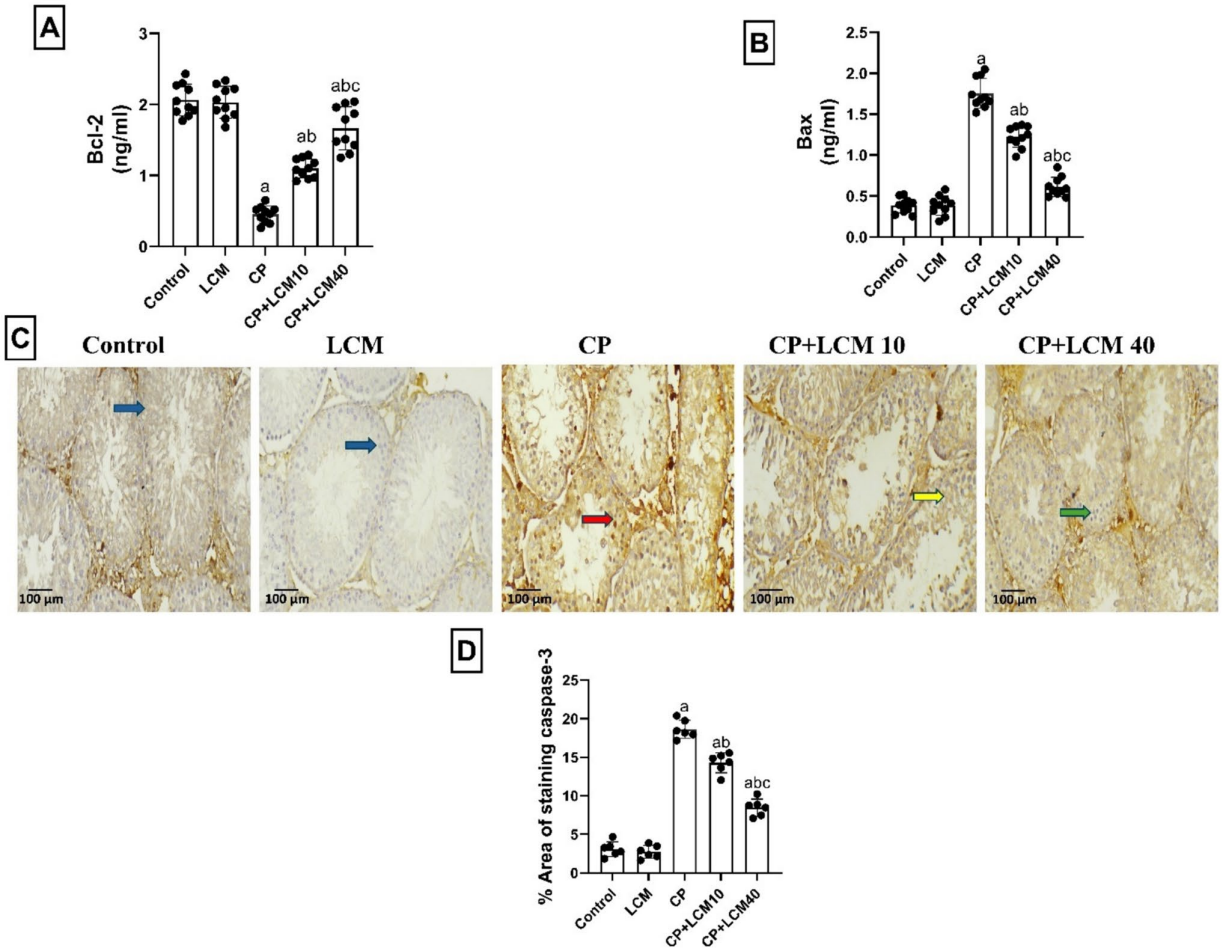
Effect of LCM on levels of apoptosis markers in testicular tissue in experimental groups. (**A**) Bcl-2; (**B**) Bax. (**C**) Representative testicular caspase-3 immunohistochemical staining photomicrographs indicate expression of caspase-3. Positive staining was brownish yellow. Minimal expression is observed in control and LCM groups (blue arrows), with marked upregulation in CP group (red arrows). CP + LCM10 group shows intermediate expression (yellow arrows), while CP + LCM40 group demonstrates substantial downregulation (green arrows). (X 200, scale bar = 100 μm); (**D**) Area percentage of Caspase-3 immunohistochemical expression. The Data are expressed using bar plot with the mean ± SD, (*n* = *10* for Bcl-2 and Bax assays and *n* = *6* for caspase-3 immunohistochemical assay); In comparison to the control group, the CP group, and the CP + LCM10 group, the corresponding *p*-values are less than 0.05 (^a^, ^b^, and ^c^, respectively). Bcl-2, B-cell lymphoma 2; Bax, Bcl-2-associated x protein; CP, cyclophosphamide; LCM, lacosamide

By using CP, the level of anti-apoptotic marker, Bcl-2, was significantly diminished in the CP-treated group by 78.1% (*p* < 0.0001) relative to the control animals. This level was markedly elevated in rats of CP + LCM10 and CP + LCM40 groups than in the rats received CP by 58.86% and 72.79% (*p* < 0.0001 for both), respectively, compared with the CP group. In contrast, marked elevation of apoptotic marker, Bax by 78% (*p* < 0.0001), was observed in the testicular tissue of CP rats compared with normal control animals. On the contrary, rats in the CP + LCM10 and CP + LCM40 groups demonstrated a significant reduction by 30.2% and 65.1% in Bax (*p* < 0.0001 for both), respectively, in comparison with the CP group, as represented in Fig. 9A–B.

According to immunohistochemical imaging of caspase-3, the intensity of caspase-3 staining is very high in testicular sections from rats of the CP group (*p* < 0.0001); inversely, the CP + LCM10 showed a lower intensity than the CP group (*p* < 0.0001). Moreover, the CP + LCM40 significantly lowered caspase-3 expression than the CP and CP + LCM10 groups (*p* < 0.0001) (Fig. 9C–D).

### Effect of LCM on STAT-3, JNK1, and HO-1

**Fig. 10:**
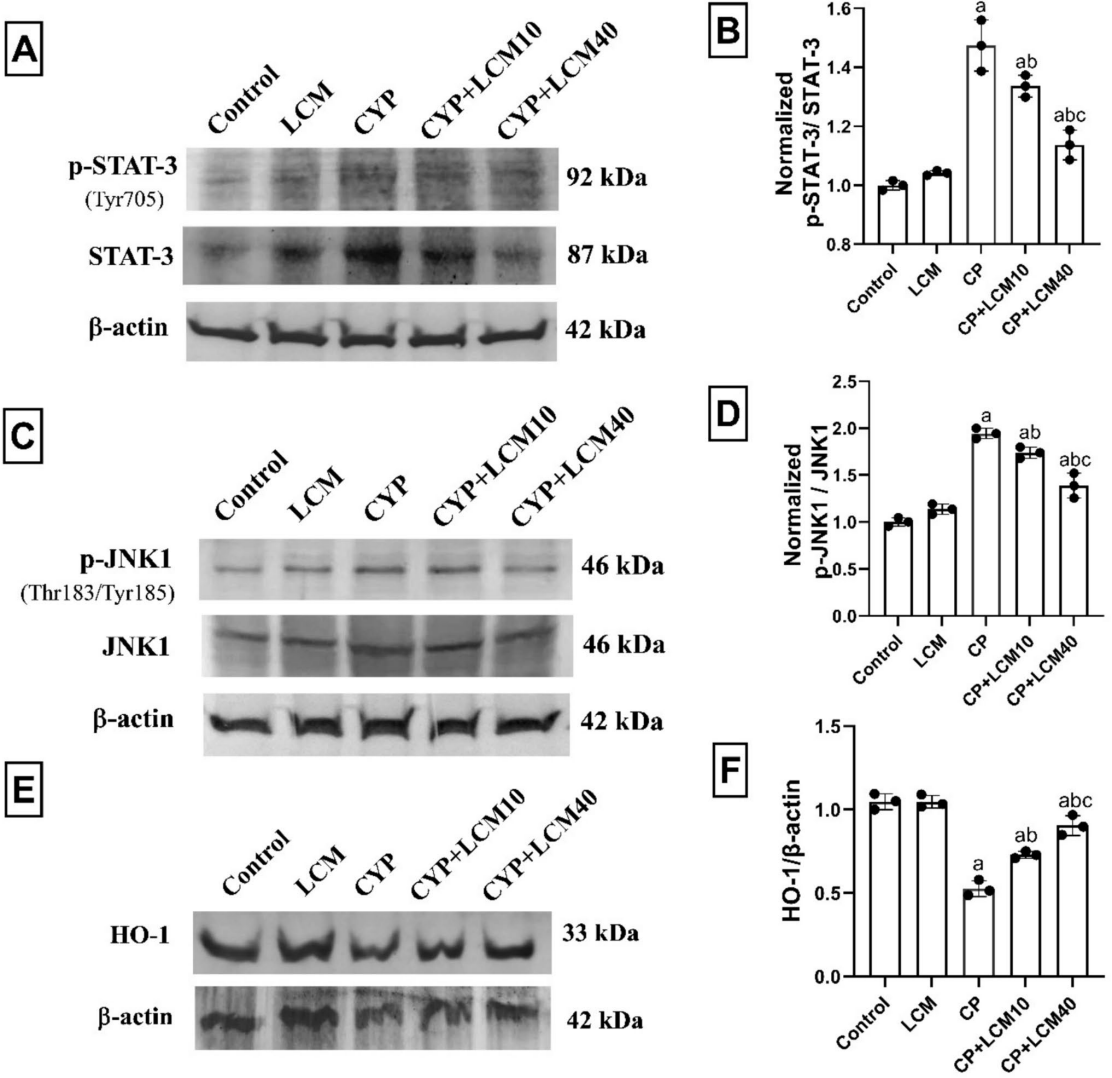
Effect of LCM on STAT-3, JNK1, and HO-1 signaling in testicular tissue in experimental groups. (**A**) Western blot bands of STAT-3 and p-STAT-3; (**B**) Densitometric data of p-STAT-3/STAT-3; (**C**) Western blot bands of JNK1 and p-JNK1; (**D**) Densitometric data of p-JNK1/JNK1; (**E**) Western blot bands of HO-1; (**F**) Densitometric data of HO-1/β-Actin. The bars demonstrate the mean ± SD, (*n* = *3*); In comparison to the control group, the CP group, and the CP + LCM10 group, the corresponding *p*-values are less than 0.05 (^a^, ^b^, and.^c^, respectively). CP, cyclophosphamide; Heme oxygenase 1, HO-1; JNK1, c-jun N-terminal kinase 1; LCM, lacosamide; p-JNK1, phosphor-c-jun N-terminal kinase 1; p-STAT-3, phosphor-signal transducer and activator of transcription 3; STAT-3, Signal transducer and activator of transcription 3

Statistical analysis of western blotting results showed that p-STAT-3/STAT-3 and p-JNK1/JNK1 ratio increased by 32.15% (*p* < 0.0001) and 48.52% (*p* < 0.0001), while HO-1/β-Actin ratio was significantly decreased by 62.6% (*p* < 0.0001) in CP group, respectively, with respect to the values observed in control rats. Compared to the CP-treated group, the CP + LCM10 and CP + LCM40 groups showed substantially reduced expression of these proteins, as follows: p-STAT-3/STAT-3: 9.4% and 22.9% (*p* = 0.0376 and *p* < 0.0001), and p-JNK1/JNK1: 10.6% and 28.6% (*p* = 0.0493 and *p* < 0.0001), respectively, with increased HO-1/β-Actin: 48.76% and 59.75% (*p* = 0.0018 and *p* < 0.0001), respectively (Fig. 10A–F).

### Correlation analysis between parameters and group comparison patterns

**Fig. 11:**
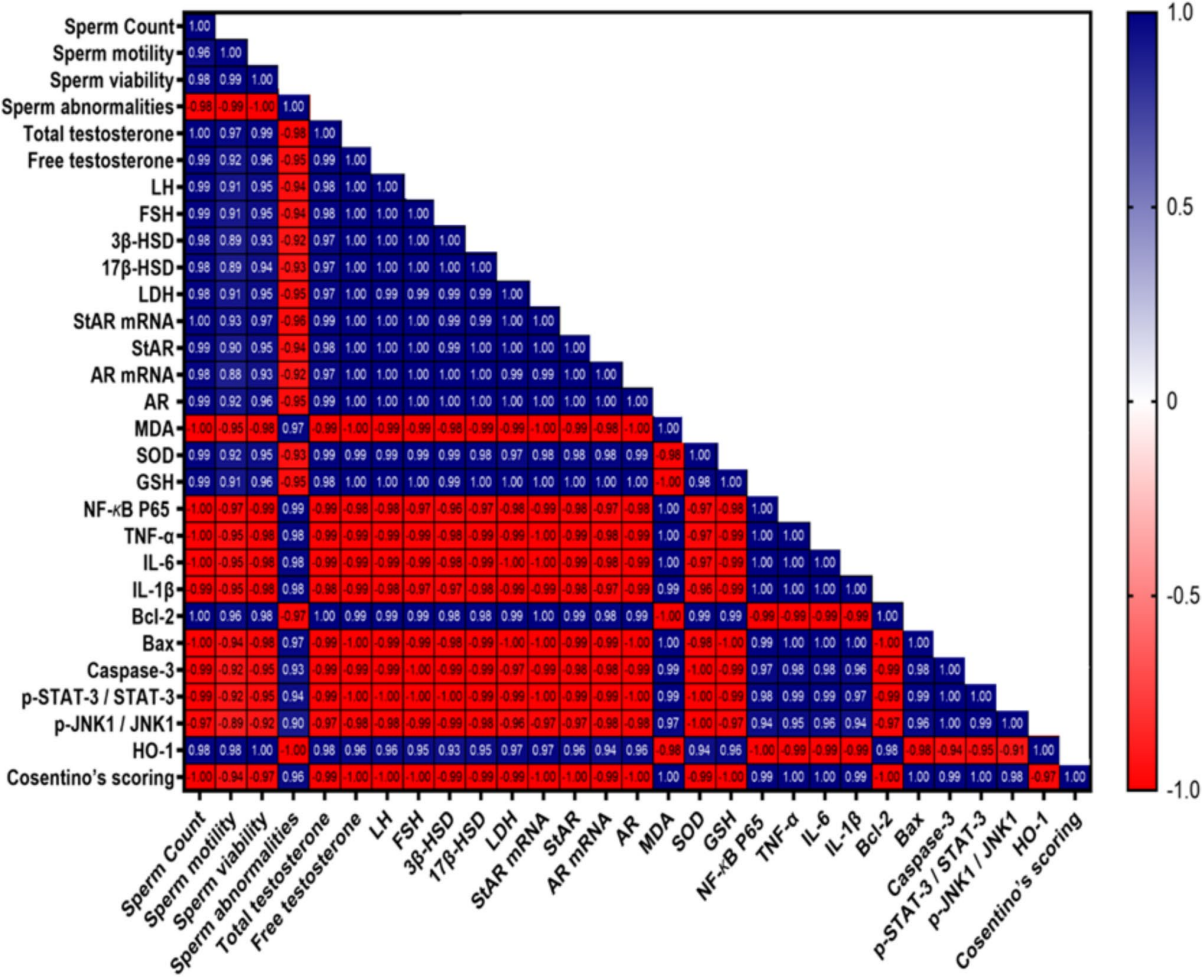
Correlation analysis heatmap showing interconnections among all measured parameters in experimental groups. Correlation was tested between different parameters using Pearson correlation. Blue color reflect positive correlation between parameters and correlation coefficient r is displayed in the figure in each box. All correlations had p < 0.05. Color scale represents correlation coefficients from −1.0 (red, strong negative correlation) to + 1.0 (blue, strong positive correlation). AR, androgen receptor; Bax, BCL2 associated X; Bcl-2, B-cell lymphoma 2; CP, cyclophosphamide; FSH, follicle-stimulating hormone; GSH, glutathione; HO-1, heme oxygenase 1; IL, interleukin; JNK1, c-jun N-terminal kinase 1; LCM, lacosamide; LDH, lactate dehydrogenase; LH, luteinizing hormone; MDA, malondialdehyde; NF-*κ*B, nuclear factor kappa B; p-JNK1, phospho-c-jun N-terminal kinase 1; p-STAT-3, phospho-signal transducer and activator of transcription 3; SOD, superoxide dismutase; StAR, steroidogenic acute regulatory protein; STAT-3, signal transducer and activator of transcription 3; TNF-α, tumor necrosis factor alpha; 3β-HSD, 3β-hydroxysteroid dehydrogenase; 17β-HSD, 17β-hydroxysteroid dehydrogenase

**Fig. 12:**
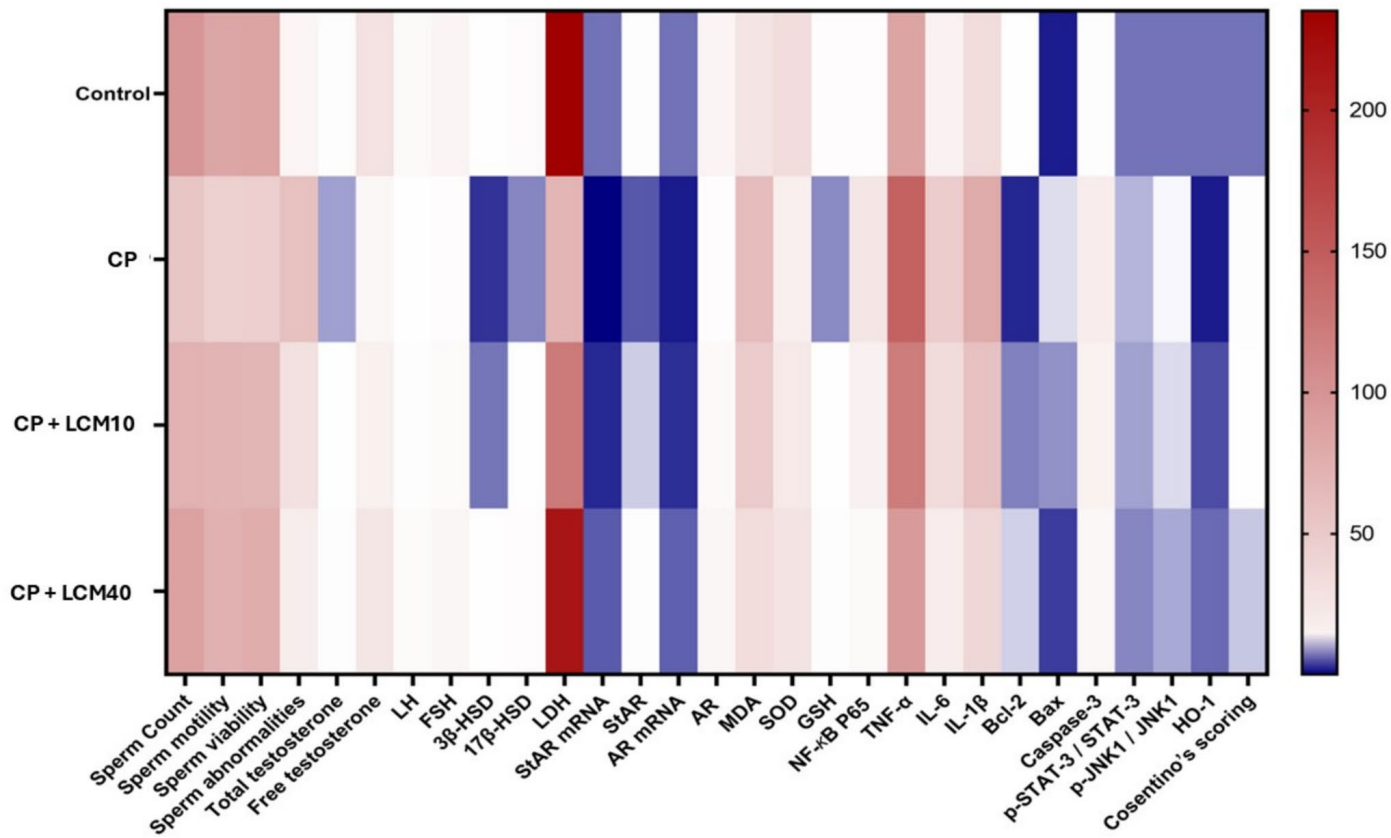
Group comparison heatmap analysis. The heatmap shows clear therapeutic pattern following: Control > CP + LCM40 > CP + LCM10 > CP for beneficial parameters, while damage markers display inverse relationship (CP > CP + LCM10 > CP + LCM40 > Control). Color scale represents normalized parameter values from minimum (blue) to maximum (red). AR, androgen receptor; Bax, BCL2 associated X; Bcl-2, B-cell lymphoma 2; CP, cyclophosphamide; FSH, follicle-stimulating hormone; GSH, glutathione; HO-1, heme oxygenase 1; IL, interleukin; JNK1, c-jun N-terminal kinase 1; LCM, lacosamide; LDH, lactate dehydrogenase; LH, luteinizing hormone; MDA, malondialdehyde; NF-*κ*B, nuclear factor kappa B; p-JNK1, phospho-c-jun N-terminal kinase 1; p-STAT-3, phospho-signal transducer and activator of transcription 3; SOD, superoxide dismutase; StAR, steroidogenic acute regulatory protein; STAT-3, signal transducer and activator of transcription 3; TNF-α, tumor necrosis factor alpha; 3β-HSD, 3β-hydroxysteroid dehydrogenase; 17β-HSD, 17β-hydroxysteroid dehydrogenase

As illustrated in Fig. 11, Pearson correlation analysis revealed distinct clustering patterns among measured parameters, with strong positive correlations observed between sperm quality parameters (count, motility, viability: r = 0.96–0.99), hormonal factors (testosterone, LH, FSH: r = 0.98–1.00), and steroidogenic enzymes (StAR, 3β-HSD, 17β-HSD: r = 0.97–1.00). Conversely, damage markers including oxidative stress (MDA), inflammatory mediators (NF-*κ*B p65, TNF-α, IL-6, IL-1β), and apoptotic factors (Bax, caspase-3) demonstrated strong negative correlations with protective parameters (r > −0.90). Mechanistic findings included p-JNK1/p-STAT-3 crosstalk (r = 0.99), validating their identification as cooperative markers in testicular injury, HO-1 emergence as a central protective hub with strong correlations to all beneficial parameters (r > 0.94), and AR functionality preservation showing critical correlation with steroidogenic enzymes (r > 0.99). Group comparison heat map analysis that represented in Fig. 12, demonstrated clear dose-dependent treatment effects, with the therapeutic pattern following: Control > CP + LCM40 > CP + LCM10 > CP across all beneficial parameters, while damage markers showed the inverse relationship (CP > CP + LCM10 > CP + LCM40 > Control). The tight clustering of sperm parameters suggests that any improvement in one aspect of sperm quality through LCM treatment will likely improve all aspects, making LCM a comprehensive protective agent with significant clinical implications for male fertility preservation during chemotherapy.

## Discussion

**Fig. 13:**
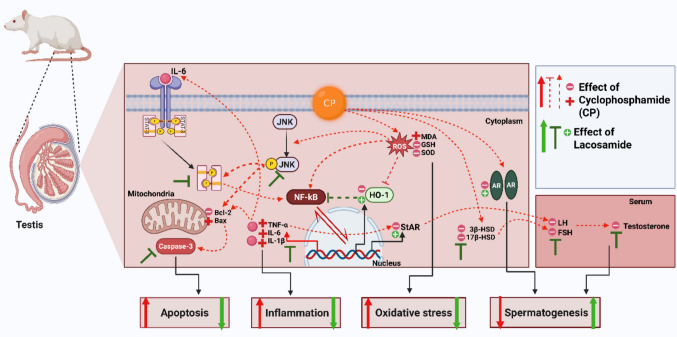
Demonstration of the possible protective mechanisms of lacosamide against testicular damage produced by cyclophosphamide in rats. AR, androgen receptor; Bcl-2, B-cell lymphoma 2; Bax, Bcl-2-associated x protein; CP, cyclophosphamide; FSH, follicle-stimulating hormone; GSH, reduced glutathione; IL-6 & 1β: Interleukin 6 & 1β; JNK1, c-jun N-terminal kinase 1; LH, luteinizing hormone; MDA, malondialdehyde; NF-*κ*B, Nuclear factor kappa B; HO-1, Heme oxygenase 1; ROS, Reactive Oxygen Species; SOD, superoxide dismutase; TNF-α, Tumor necrosis factor; STAT-3, Signal transducer and activator of transcription 3; StAR, steroidogenic acute regulatory protein, 3β-HSD, 3β-hydroxysteroid dehydrogenase; 17β-HSD, 17β-hydroxysteroid dehydrogenase

This study demonstrates lacosamide (LCM)'s protective potential against cyclophosphamide (CP)-induced testicular dysfunction, addressing a critical therapeutic challenge in cancer patients requiring concurrent antiepileptic therapy. Unlike first and second-generation antiepileptic drugs that cause testicular injury (Al Snafi et al. 2013; Eklioglu and Ilgin 2022). LCM provides comprehensive protection through novel mechanisms. We report for the first time: (1) LCM's protection against JNK1-induced apoptosis with HO-1 upregulation, (2) CP-induced STAT-3 expression in testicular tissue with p-JNK1-mediated STAT-3 activation, and (3) LCM's preservation of androgen receptor (AR) functionality against CP-induced downregulation. LCM restored oxidative balance, suppressed inflammation and apoptosis, preserved steroidogenesis, and enhanced sperm quality (Fig. 13), with correlation analysis validating its multi-target protective approach.

In the current investigation, CP administration significantly altered LH and FSH secretion relative to controls. LH promotes Leydig cells to synthesize testosterone, whereas FSH interacts with Sertoli cells that support sperm growth and maturation (Oduwole et al. 2021). This hormonal dysregulation reflects impaired Sertoli-Leydig cell functional maturation, resulting in suppressed testosterone biosynthesis and disrupted spermatogenesis (Ramaswamy and Weinbauer 2014). consistent with previous observations by Salama et al. in CP-induced testicular damage (Salama et al. 2020). We documented substantial reductions in steroidogenesis-related enzymes 3β-HSD and 17β-HSD, which directly contributed to declining sex hormone levels (Penning 2003). Additionally, CP downregulated AR, consistent with cadmium's negative impact on testicular AR in previous studies (Abdelrazek et al. 2016), LDH produces ATP through glycolysis in germ cells (Riera et al. 2001). These comprehensive findings support Salama et al., who reported that CP significantly reduced 3β-HSD, 17β-HSD, and LDH activity, decreased serum testosterone, LH, and FSH levels, and impaired epididymal sperm quality (Salama et al. 2020).

Our data demonstrated that LCM effectively attenuated CP-induced testicular damage by significantly upregulating steroidogenesis-related enzymes (3β-HSD and 17β-HSD) and enhancing StAR expression, collectively restoring sex hormone biosynthesis including LH, FSH, and testosterone. Furthermore, LCM administration markedly elevated testicular LDH. LCM-mediated upregulation of AR improved testicular function, correlating with substantial improvements in epididymal sperm quality parameters.

The biological pathogenesis of these findings is attributed to CP-induced oxidative stress. Our data demonstrated that CP administration precipitated significant ROS elevation coupled with marked depletion of endogenous antioxidant defenses. This was evidenced by increased testicular MDA, a lipid peroxidation marker, with concomitant reductions in GSH and SOD compared to controls. This imbalance resulted from accumulation of highly reactive free radicals, a consequence of CP's toxic metabolite acrolein, which causes significant oxidative stress (Liu et al. 2012a). Elevated ROS promoted NF-κB activation and expression in CP-treated rats. NF-*κ*B functions as a transcription factor modulating pro-inflammatory mediators, which were notably increased in CP-treated rats, including TNF-α, IL-1β, and IL-6 (Gloire et al. 2006; Serasanambati and Chilakapati 2016). Our findings showed decreased Bcl-2 (anti-apoptotic factor) with increased Bax (pro-apoptotic factor) due to CP-induced testicular toxicity. The observed caspase-3 upregulation in CP-exposed testicular tissue suggests apoptotic pathway activation, related to significant TNF-α upregulation, which downregulates Bcl-2 and upregulates Bax. This imbalance triggers Bax to damage the mitochondrial membrane and, together with oxidative stress, activate caspase-3 (Menon et al. 2002; Vince et al. 2018). Our results about oxidative stress, inflammation, and apoptosis markers corroborate the findings of Almuqati in CP-induced testicular injury in rats (Almuqati 2025).

In the current study, we observed considerable upregulation and phosphorylation of JNK1 in CP-treated rats. JNK1 is a stress-activated protein kinase that mediates cellular signals regulating gene expression, cell viability, apoptosis, and differentiation (Sabapathy et al. 2004), and is implicated in testicular processes such as germ cell development (Huang et al. 2021). Our findings agree with Fadel et al., which reported JNK1 association with oxidative stress in testicular germ cells enhanced by ROS generation (Fadel et al. 2020). Additionally, the phosphorylated form of JNK1 is known to upregulate the expression of proteins essential for apoptosis, such as Caspase-3 (Saad et al. 2020). We further found considerable upregulation and phosphorylation of STAT-3, linked to significantly elevated IL-6, a well-established STAT-3 activator (Hu et al. 2021). The findings are in agreement with the prior research by Abdel-Aziz et al. in testicular ischemia/reperfusion injury in rats, which observed upregulation of the IL-6/STAT-3 pathway (Abdel-Aziz et al. 2024). Upon activation and phosphorylation, STAT-3 translocates into the nucleus, facilitating sustained NF-*κ*B transcriptional activity and upregulating inflammatory cytokine production (Lee et al. 2009; Hu et al. 2021). As a result, there is a self-perpetuating cycle where IL-6 enhances its production by activating STAT-3. Additionally, the active form of JNK1 is associated with the activation of STAT-3 (Liu et al. 2012b). Furthermore, CP-treated rats exhibited significantly decreased HO-1 expression, aligning with Wang et al., which evaluated HO-1's defense mechanism against CP-induced testicular injury (Wang et al. 2021). HO-1 exhibits antioxidant effects, suppresses inflammation, and inhibits the NF-*κ*B/IL-6 axis that activates STAT-3 (Qu et al. 2018).

We propose that LCM counteracts CP-induced toxicity by restoring cellular changes to near normal. The present investigation demonstrated substantial improvements in CP + LCM10 and CP + LCM40 groups. LCM markedly mitigated CP-induced oxidative stress, exhibiting protective effects against free radical production through increased testicular GSH and SOD and decreased MDA compared to the CP group, consistent with Demiroz et al., who investigated LCM's neuroprotective role against peripheral nerve injury in rats (Demiroz et al. 2020). Consequently, LCM prevented ROS-induced NF-*κ*B triggering and downstream pro-inflammatory cytokines (TNF-α, IL-1β, and IL-6), demonstrating anti-inflammatory properties. These results support Al-Massri et al., who explored LCM's neuroprotective and anti-inflammatory effects in paclitaxel-induced neuropathy (Al-Massri et al. 2018). The anti-inflammatory effect was also linked with HO-1 upregulation as a protective protein controlling testicular injury. Moreover, LCM effectively elevated Bcl-2 and lowered Bax and caspase-3, showing powerful anti-apoptotic impact (Haddad 2002; Menon et al. 2002). consistent with Yao et al., who studied LCM's protective effect against bilateral cavernous nerve injury-induced erectile dysfunction (Yao et al. 2024). Furthermore, LCM notably decreased the protein expression of JNK1 as well as its active phosphorylated form, and this effect was linked to the decreased levels of the abovementioned oxidative stress and apoptotic markers. Collectively, LCM showed a possible downregulation in STAT-3 and p-STAT-3 and that results are aligned with those of Al-Massri et al. STAT-3 downregulation was attributed to suppressed NF-*κ*B and IL-6, decreased p-JNK1, and upregulated AR and HO-1, which collectively abrogated inflammatory signaling (Wang et al. 2016). CP induced testicular degeneration with vacuolation, necrosis, reduced germinal epithelium, and irregular seminiferous tubules. LCM therapy significantly alleviated these histological abnormalities, consistent with Salama et al. (Salama et al. 2020). Fortunately, the previous results were alleviated, and seminiferous tubules degeneration was decreased following LCM therapy. These data suggest that LCM therapy possibly suppress oxidative stress, inflammation, and apoptosis in the testes.

Notably, LCM's ameliorative effects demonstrated a dose-dependent pattern, with the higher dose (40 mg/kg) exhibiting more pronounced protective effects than the lower dose (10 mg/kg). This dose-dependent relationship further substantiates LCM's pharmacological efficacy in mitigating CP-induced testicular damage.

Moreover, those undergoing chemotherapy treatment for their autoimmune disorders or malignancies exhibit an increased susceptibility to experiencing epileptic seizures and peripheral neuropathy (Windebank and Grisold 2008; Weller et al. 2012). Notably, LCM has demonstrated significant neuroprotective efficacy in patients experiencing epilepsy or neurotoxicity because of chemotherapy (Villanueva et al. 2016; Argyriou et al. 2020). The current study presents LCM as a promising therapeutic approach to mitigate testicular damage from cancer chemotherapy, which may be accompanied by epilepsy or neurotoxicity. This dual therapeutic potential positions LCM as a valuable agent for oncology patients, potentially addressing both chemotherapy-induced reproductive toxicity and neurological complications simultaneously.

## Conclusion

This study demonstrates that LCM, as the first drug of third-generation antiepileptic drugs investigated for gonadoprotection, effectively protects against CP-induced testicular damage through mechanisms including JNK1-induced apoptosis inhibition, HO-1-mediated IL-6/STAT-3 suppression, prevention of p-JNK1/p-STAT-3 crosstalk, and AR preservation. These findings establish LCM as a promising therapeutic breakthrough for male fertility preservation during chemotherapy.

## Supplementary Information

Below is the link to the electronic supplementary material.

Supplementary file1 (PDF 807 KB)

## Data Availability

Our data collected in this research is confidential and will be made available only on a reasonable request.
