# Supplementary material for: Lacosamide prevents cyclophosphamide-induced testicular dysfunction via inhibition of NF-κB/IL-6/STAT-3 and JNK1/Caspase-3 axes with AR and HO-1 preservation: in vivo and in silico evidence
Source: Naunyn Schmiedebergs Arch Pharmacol. 2026 Feb 2;399(7):10281–301. doi: 10.1007/s00210-026-04994-7 (PMC13152971; doi:10.1007/s00210-026-04994-7)
Supplement: Supplementary file 1 — Supplementary file1 (PDF 807 KB) [file 210_2026_4994_MOESM1_ESM.pdf]

<u>**Original Western blot for three repeats**</u>

<u>**Sample (1)**</u>

p-STAT-3 (Tyr705) (sample 1)

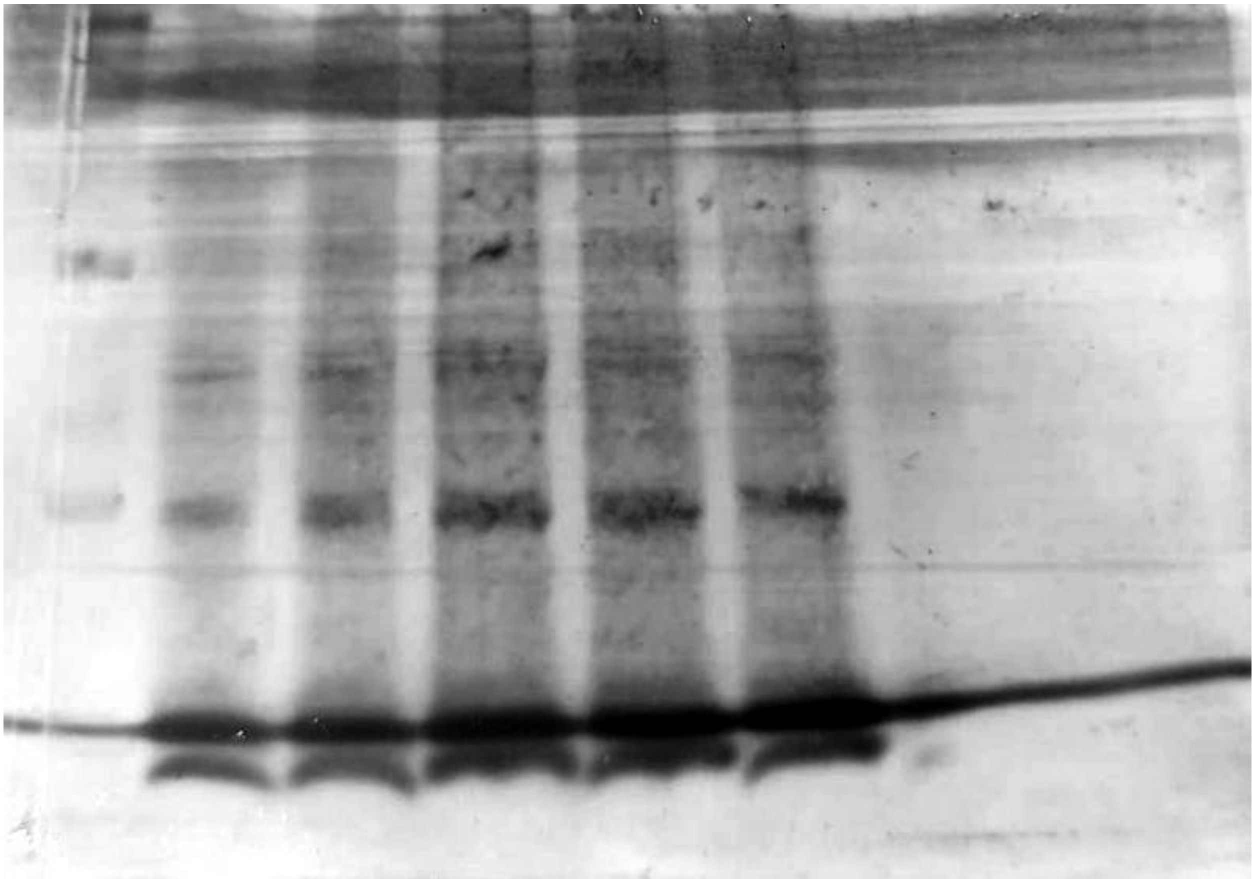

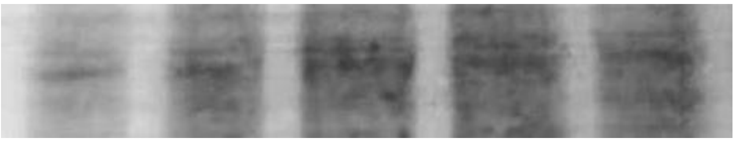

92 kDa

STAT-3 (sample 1)

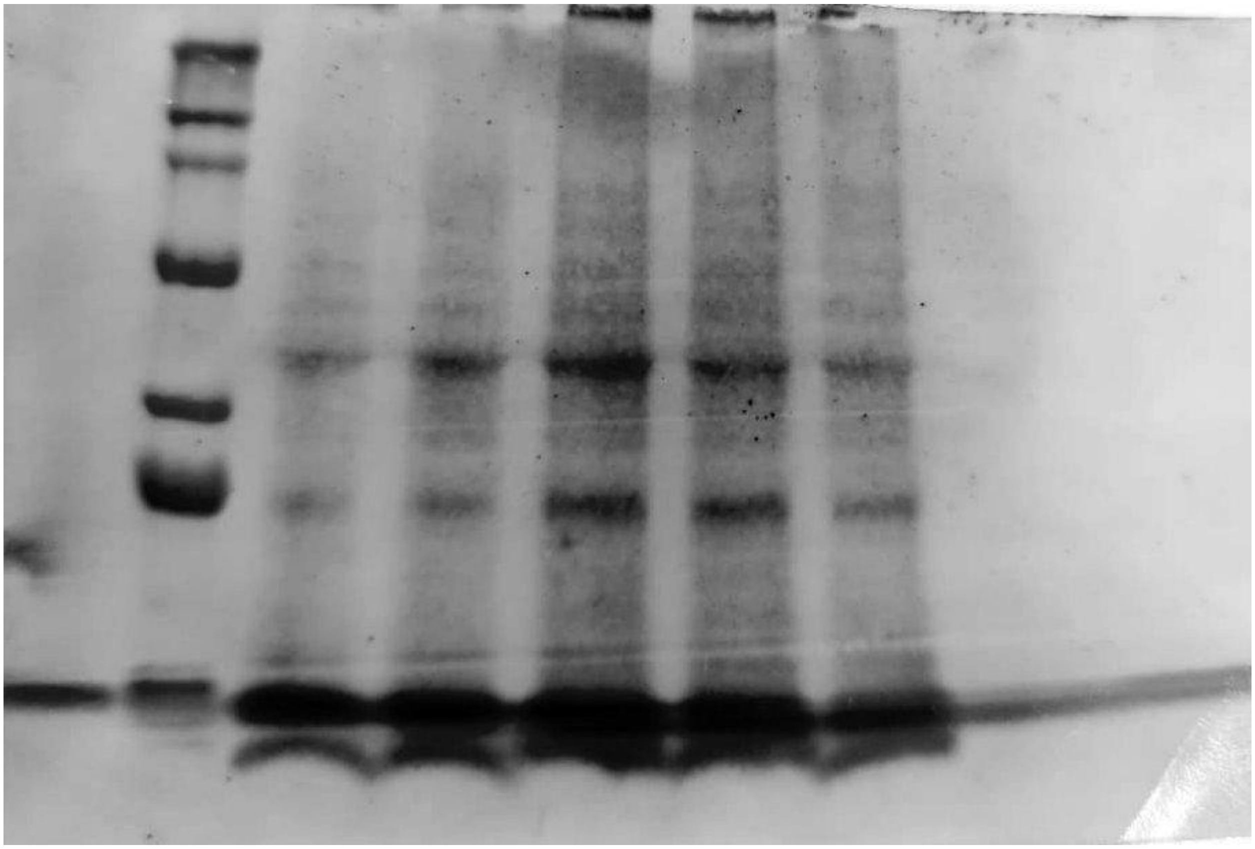

87 kDa

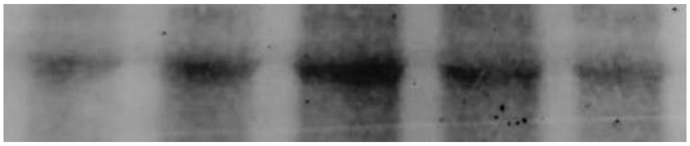

p-JNK1 (Thr183/Tyr185) (sample 1)

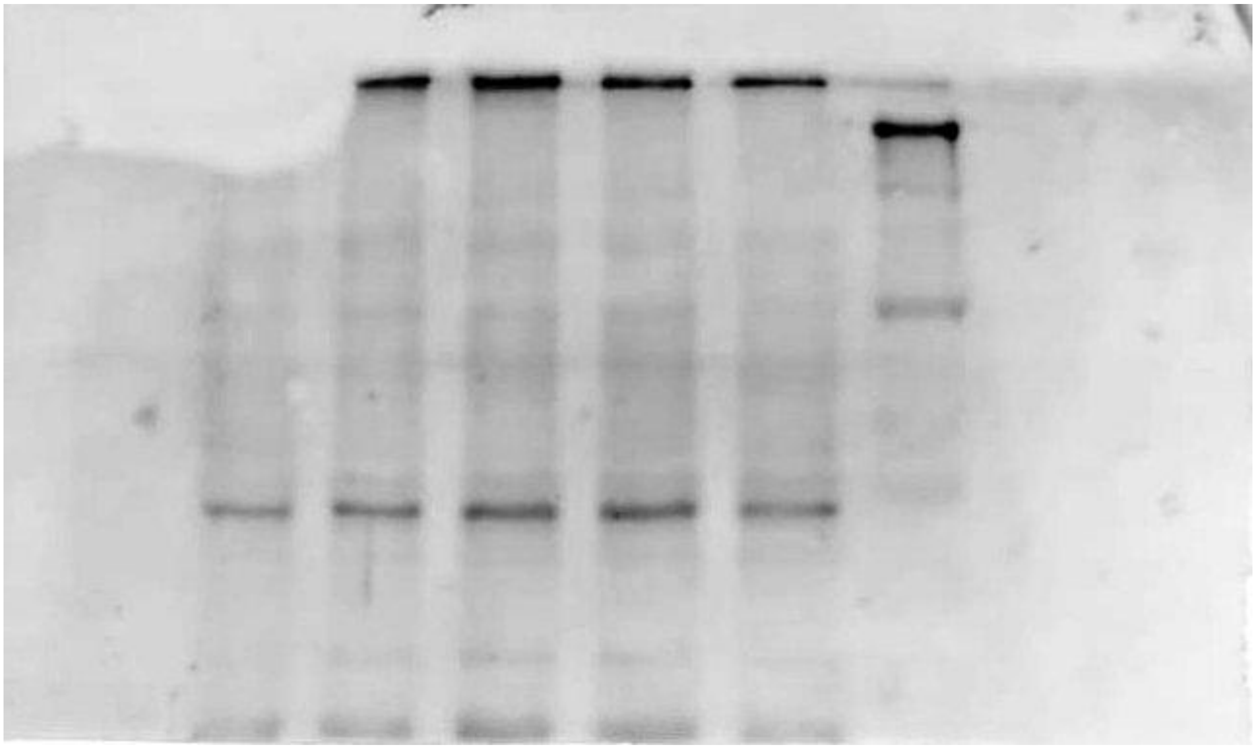

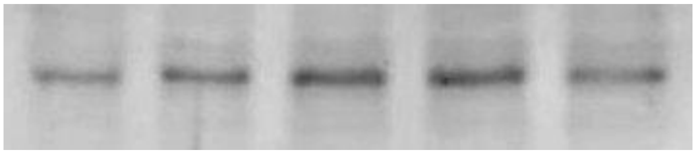

46 kDa

Total JNK1 (sample 1)

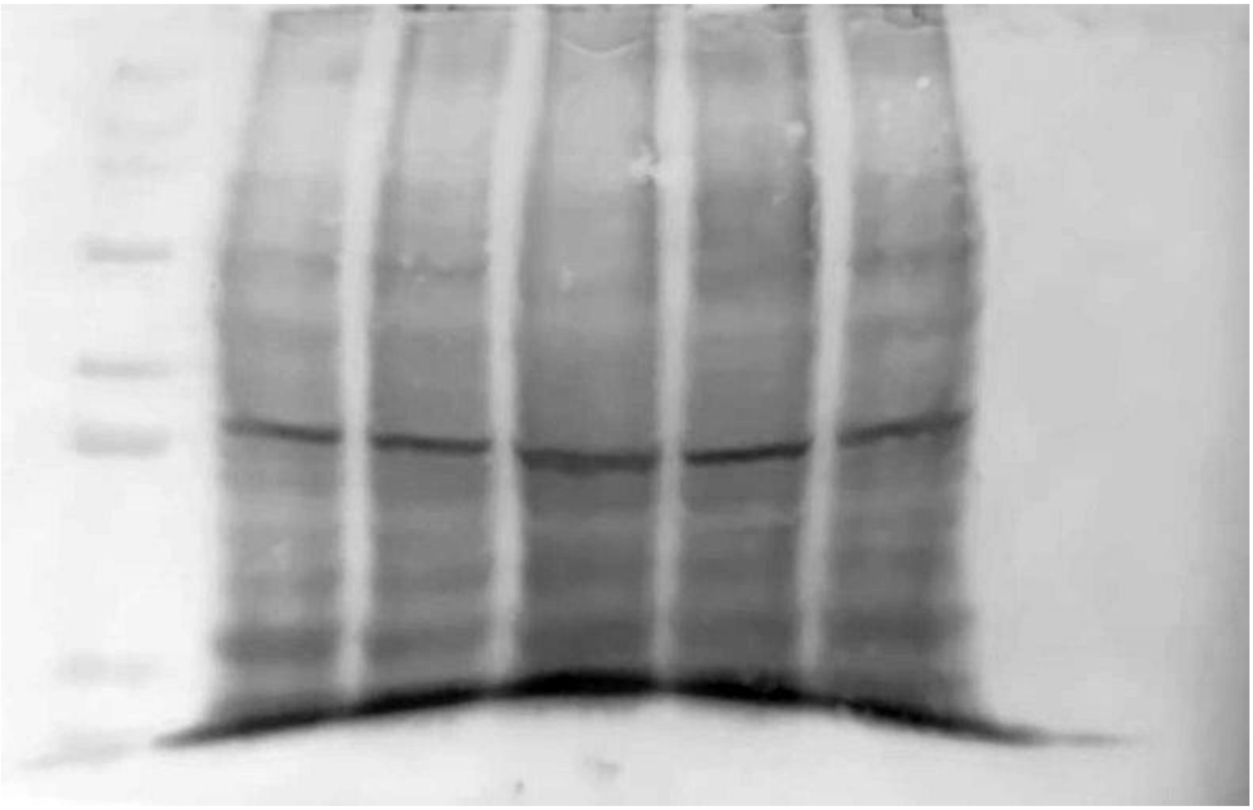

46 kDa

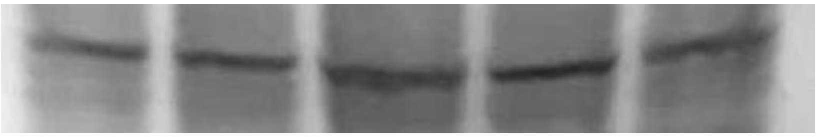

HO-1 (Sample 1)

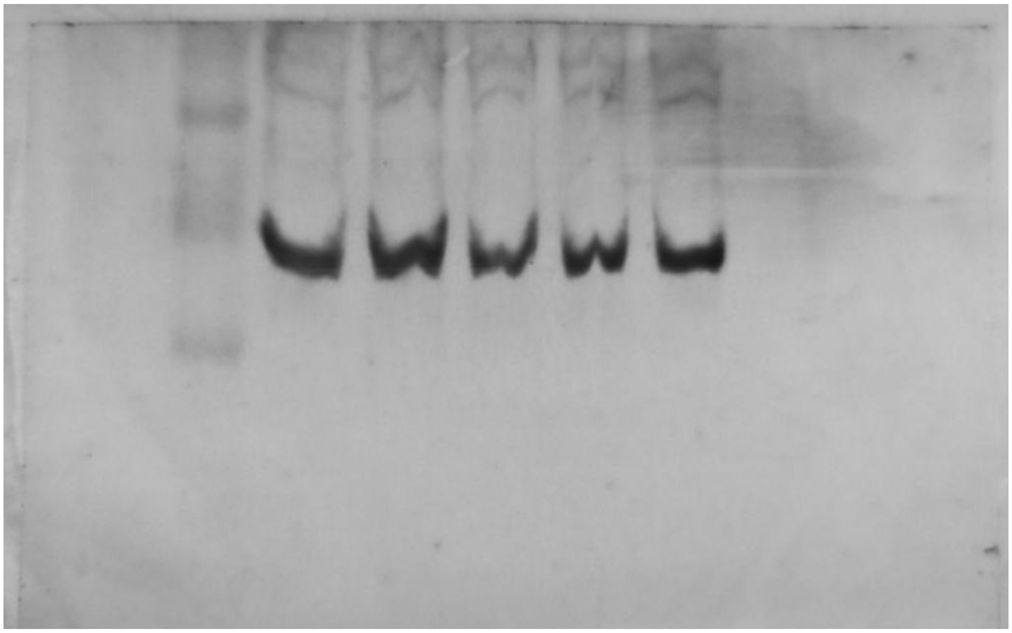

33 kDa

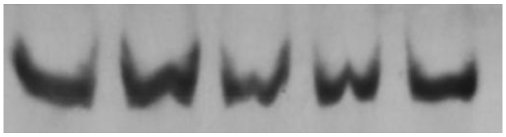

β-actin (Sample 1)

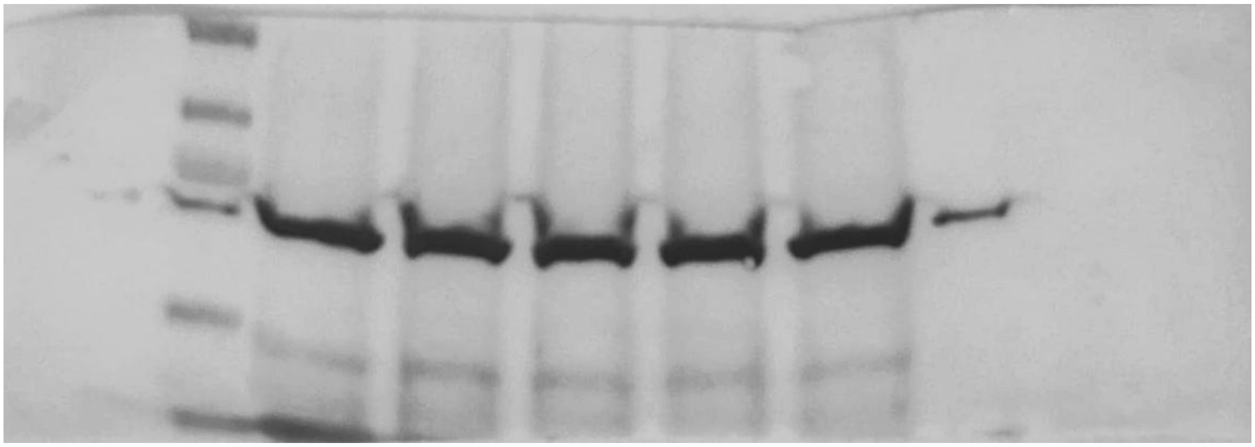

42 kDa

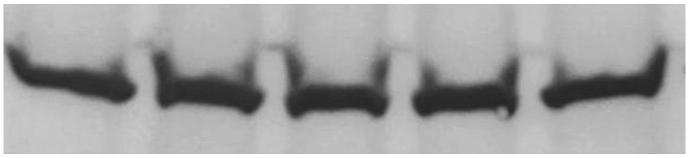

Sample 2

p-STAT-3 (Tyr705) (sample 2)

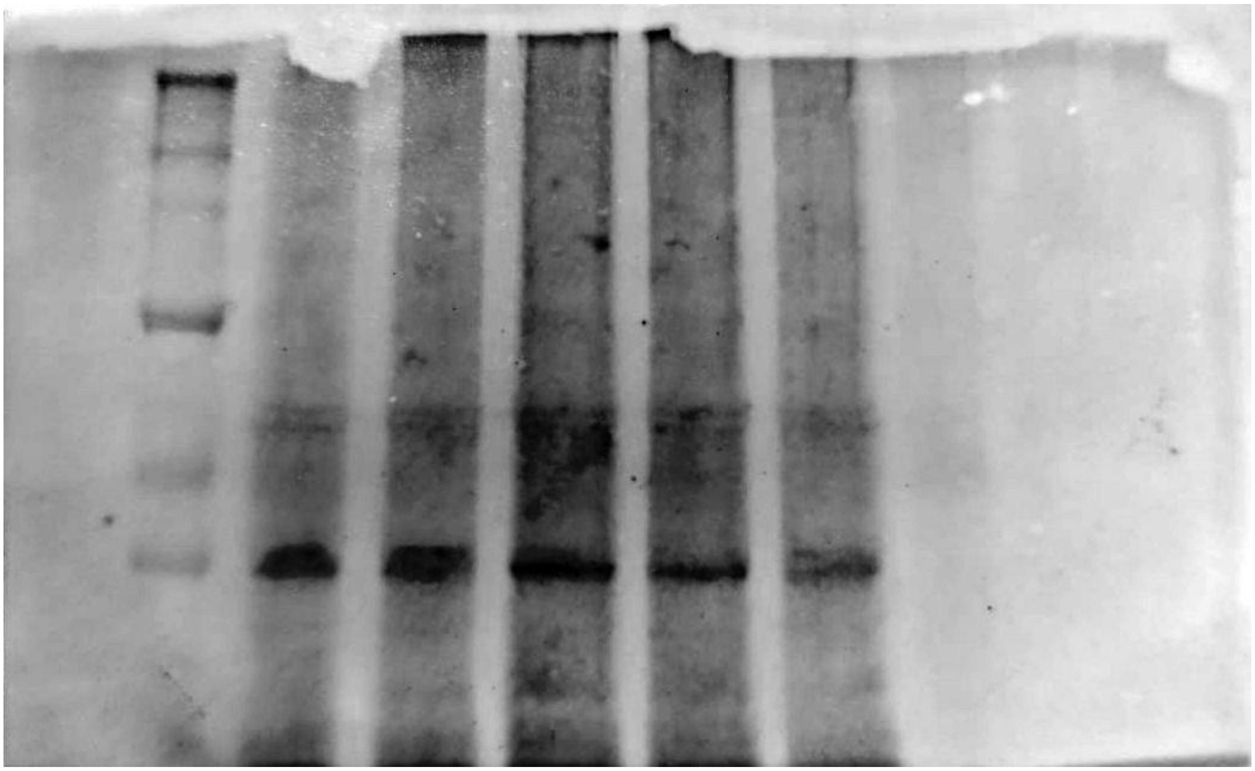

92 kDa

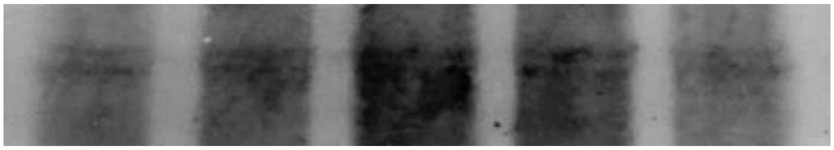

STAT-3 (sample 2)

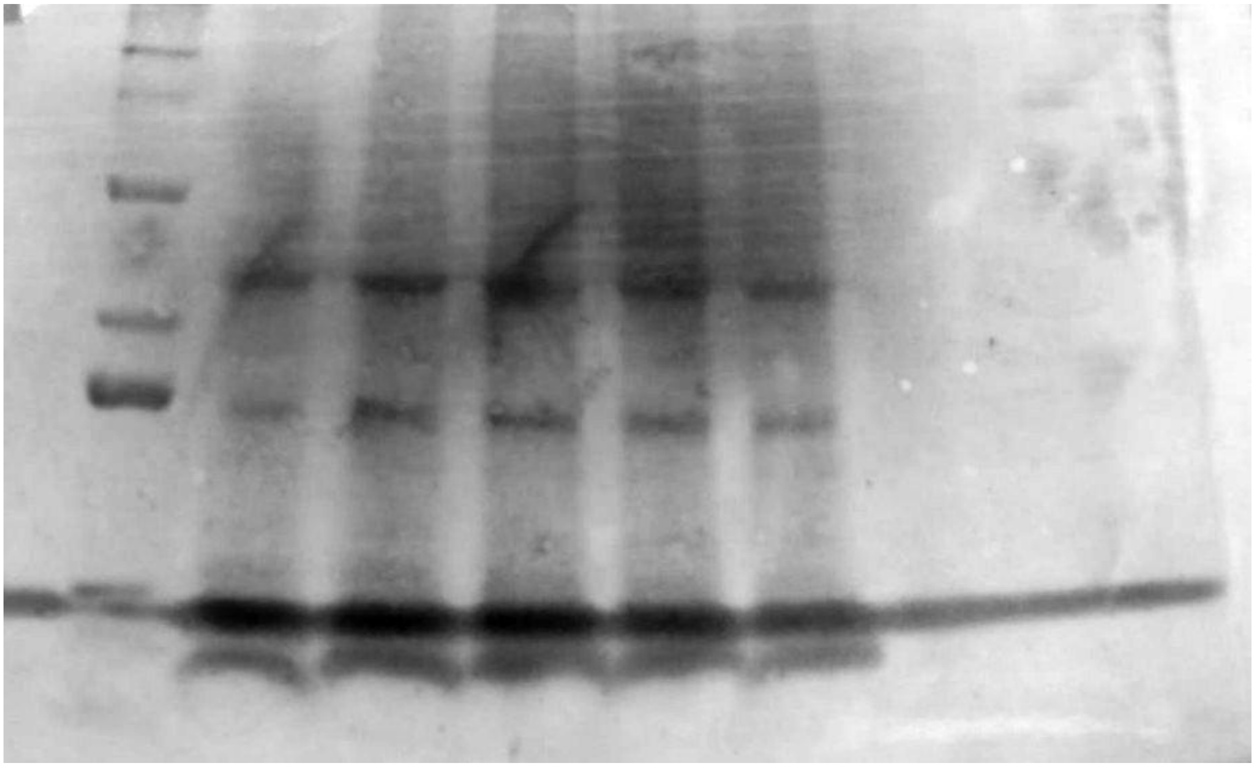

87 kDa

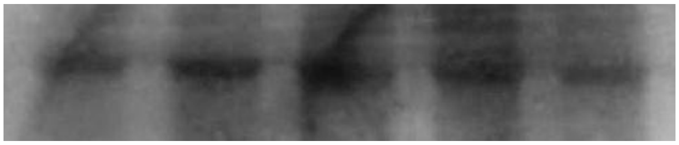

p-JNK1 (Thr183/Tyr185) (sample 2)

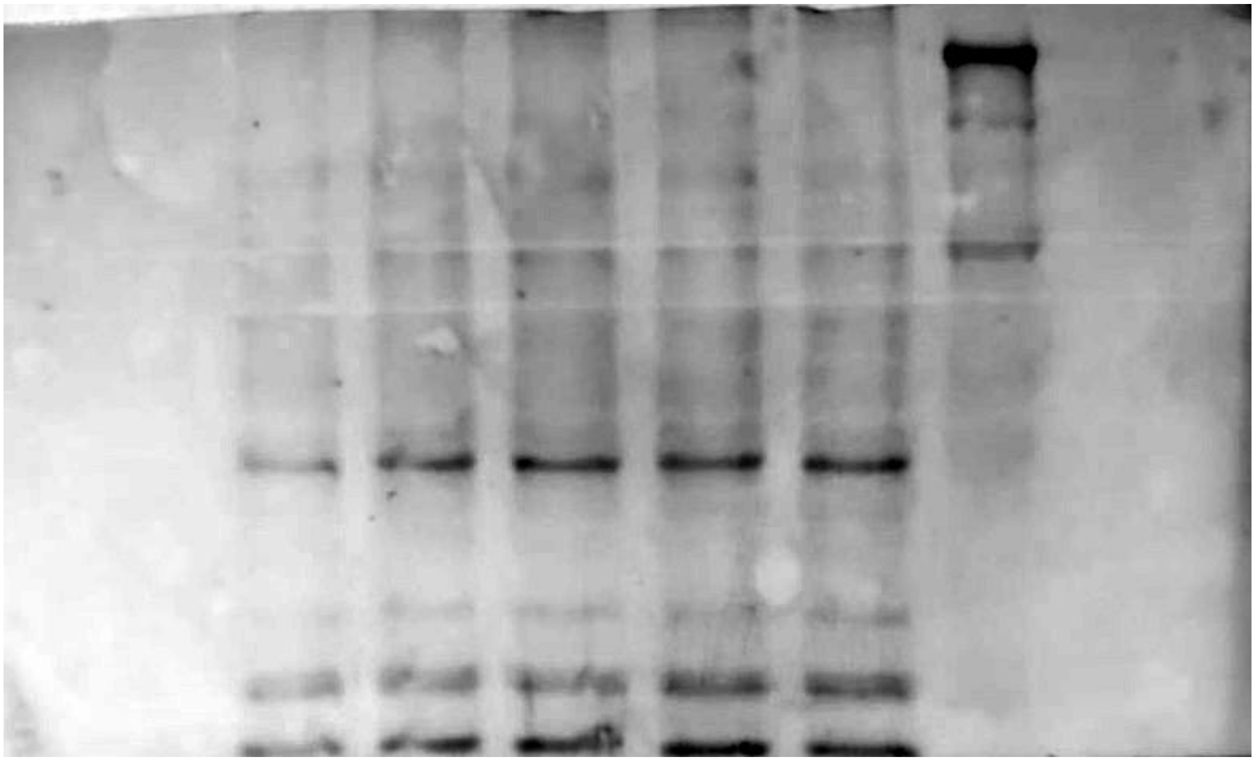

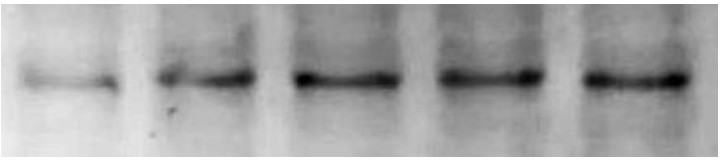

46 kDa

Total JNK1 (sample 2)

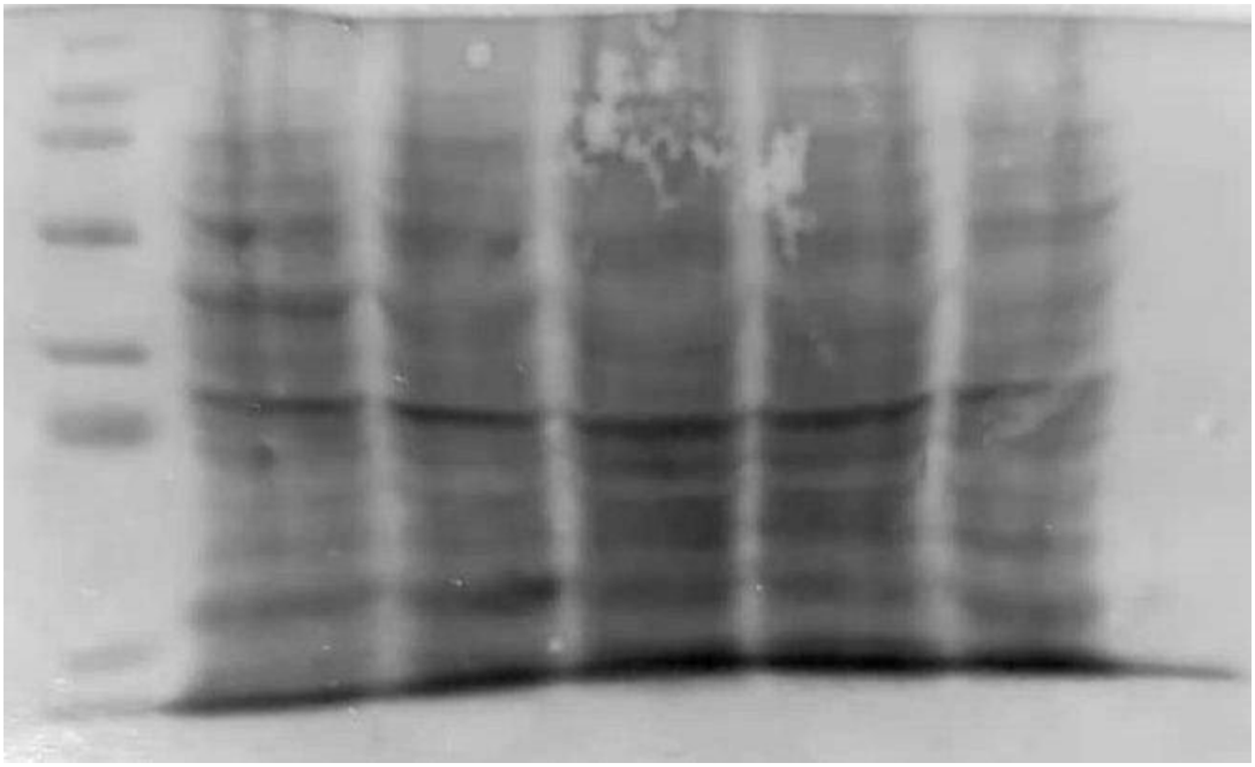

46 kDa

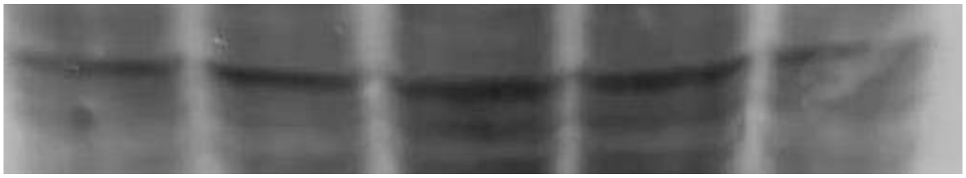

HO-1 (Sample 2)

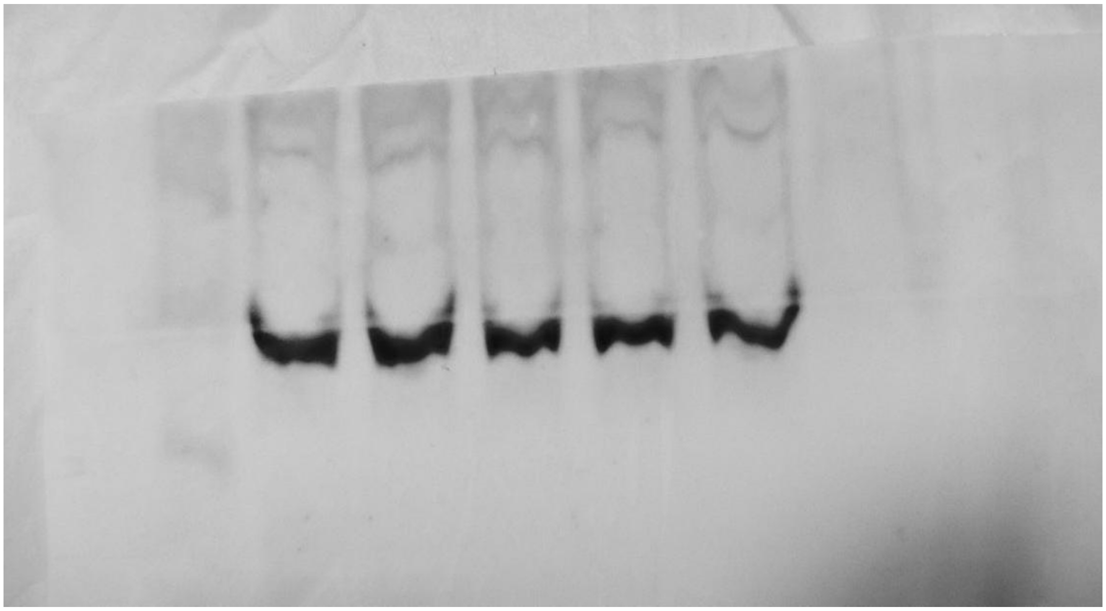

33 kDa

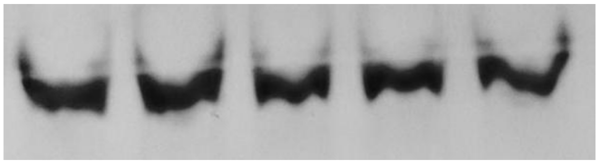

β-actin (Sample 2)

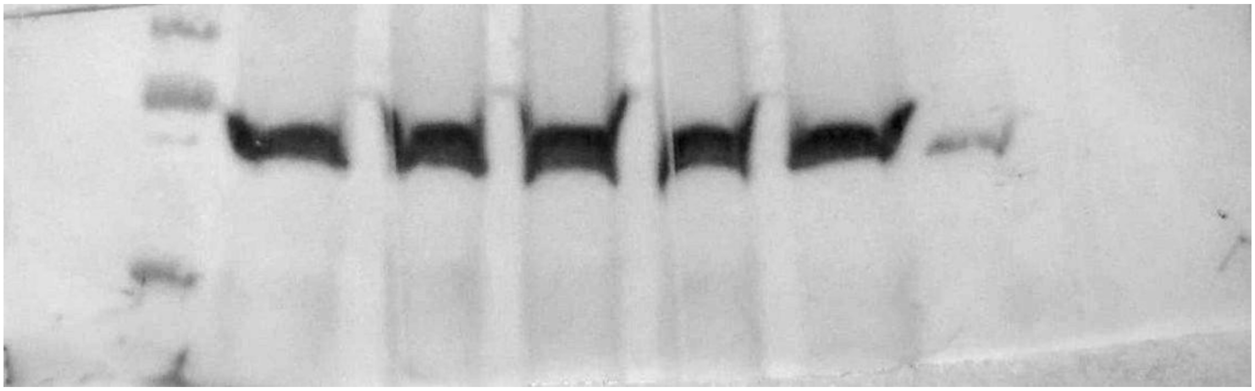

42 kDa

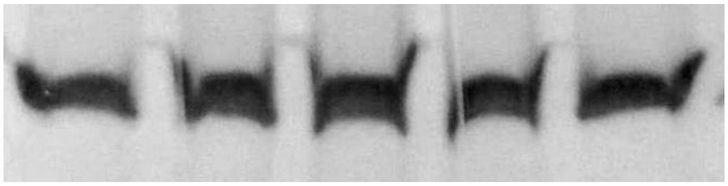

Sample (3)

p-STAT-3 (Tyr705) (sample 3)

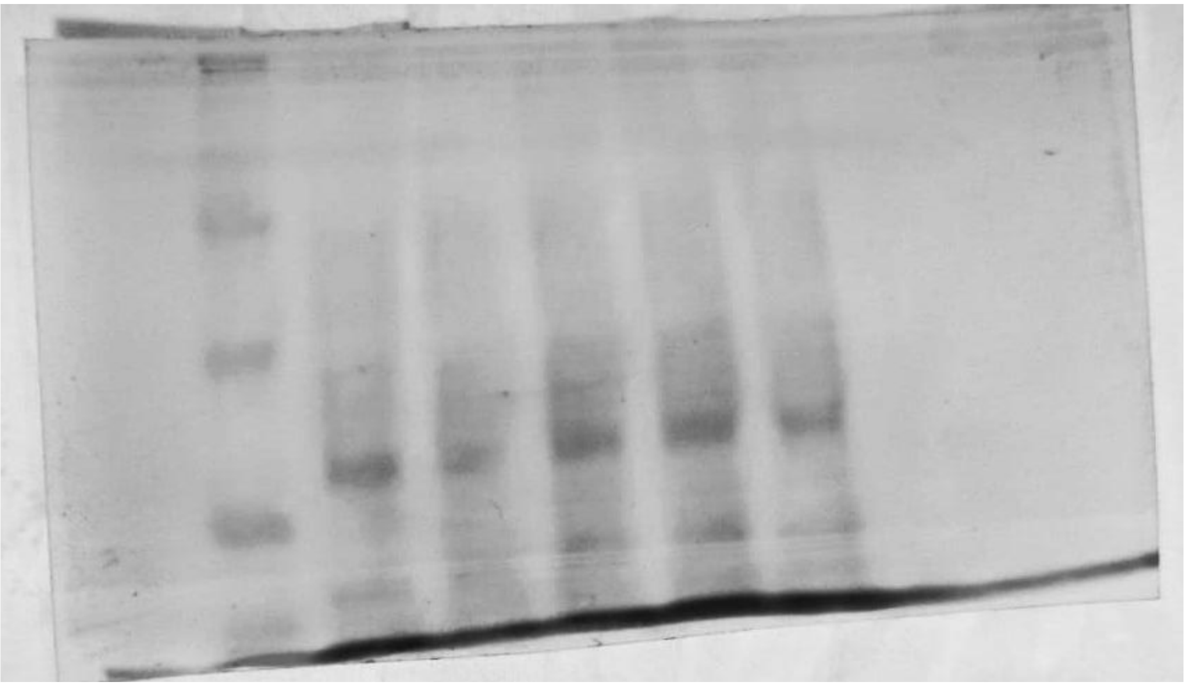

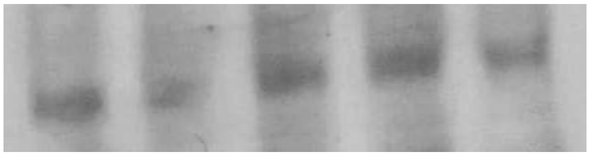

92 kDa

STAT-3 (sample 3)

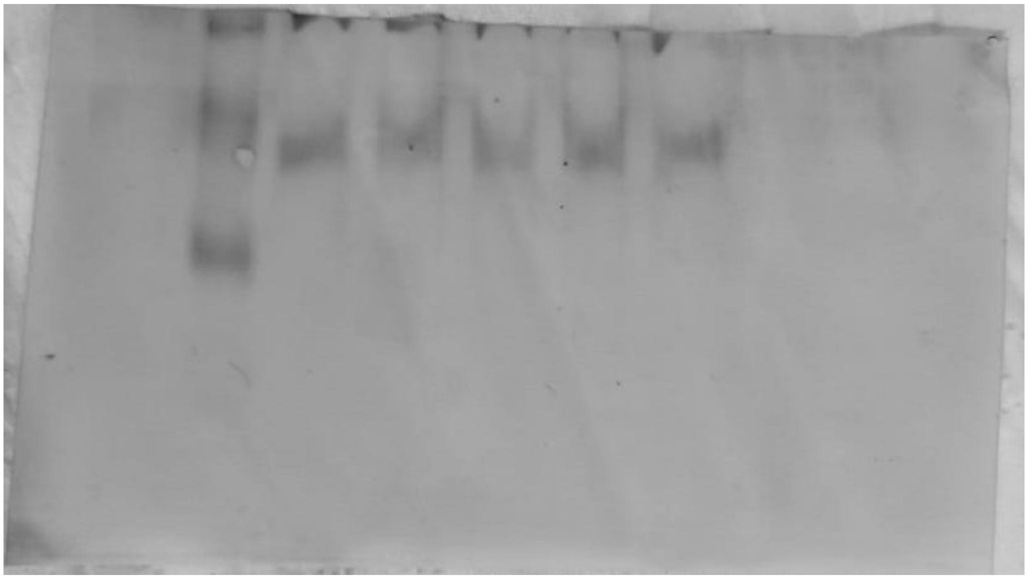

87 kDa

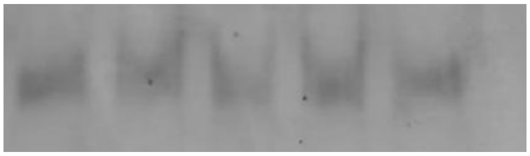

p-JNK1 (Thr183/Tyr185) (sample 3)

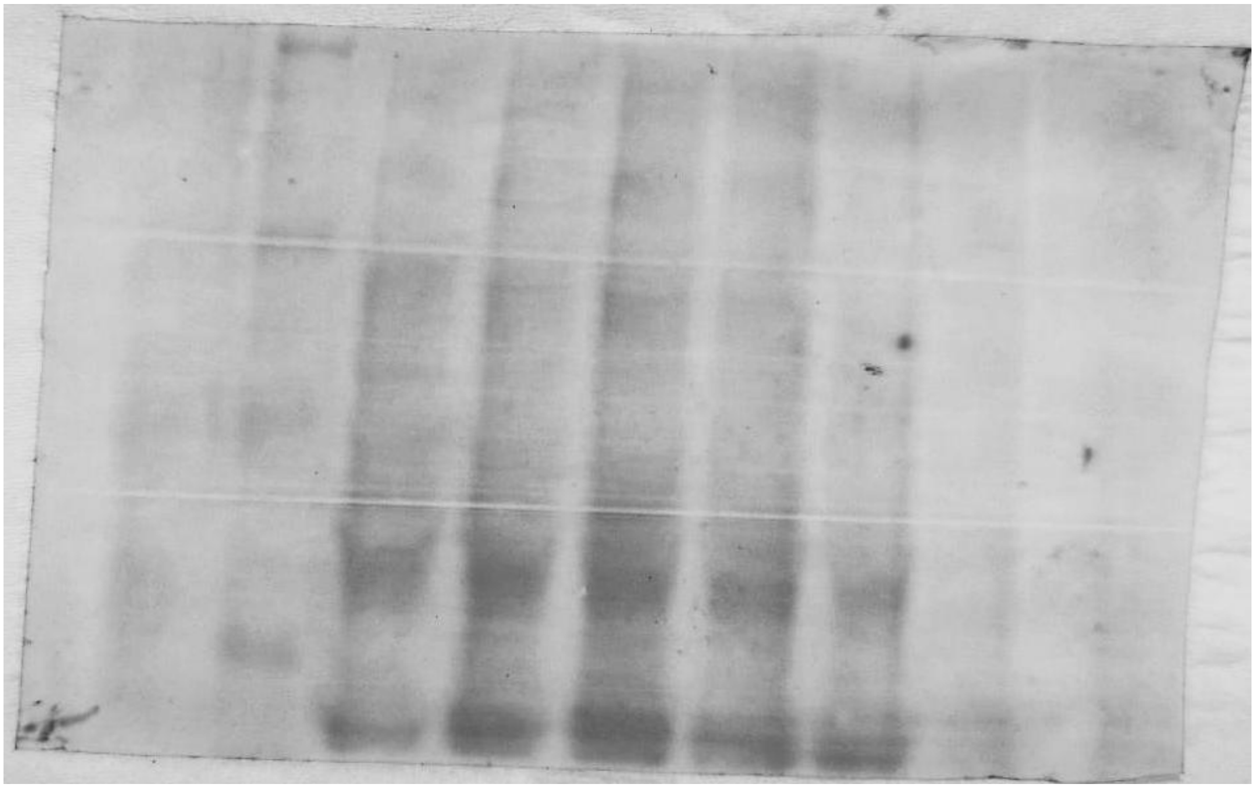

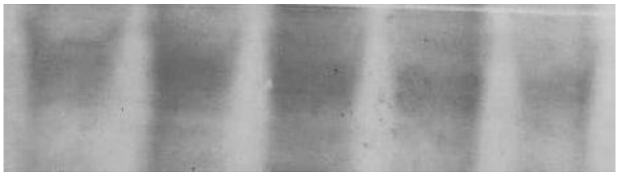

46 kDa

Total JNK1 (sample 3)

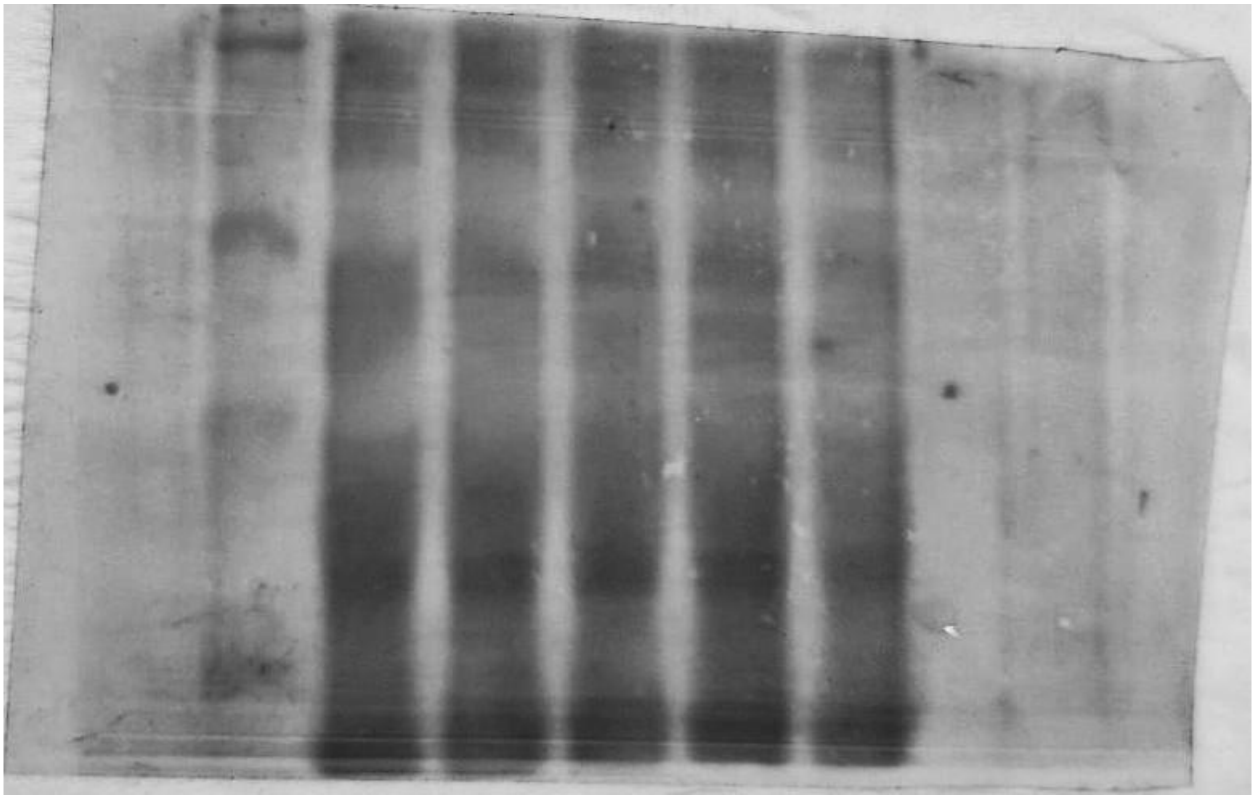

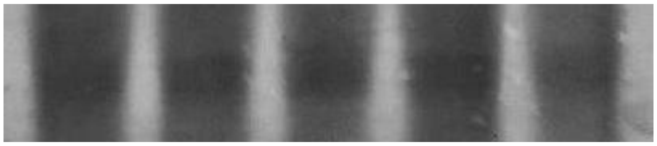

46 kDa

HO-1 (Sample 3)

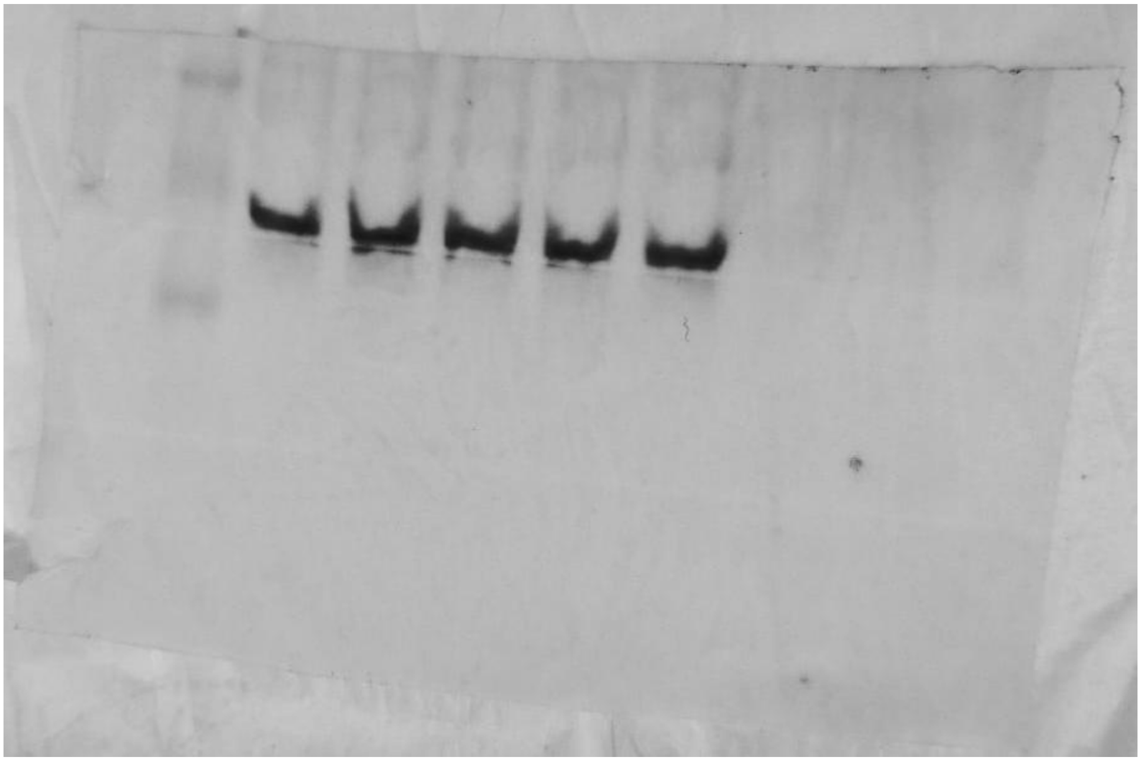

33 kDa

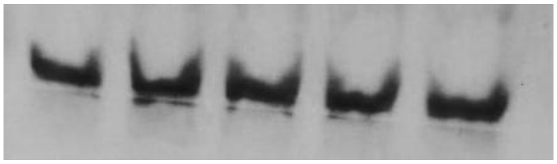

β-actin (sample 3)

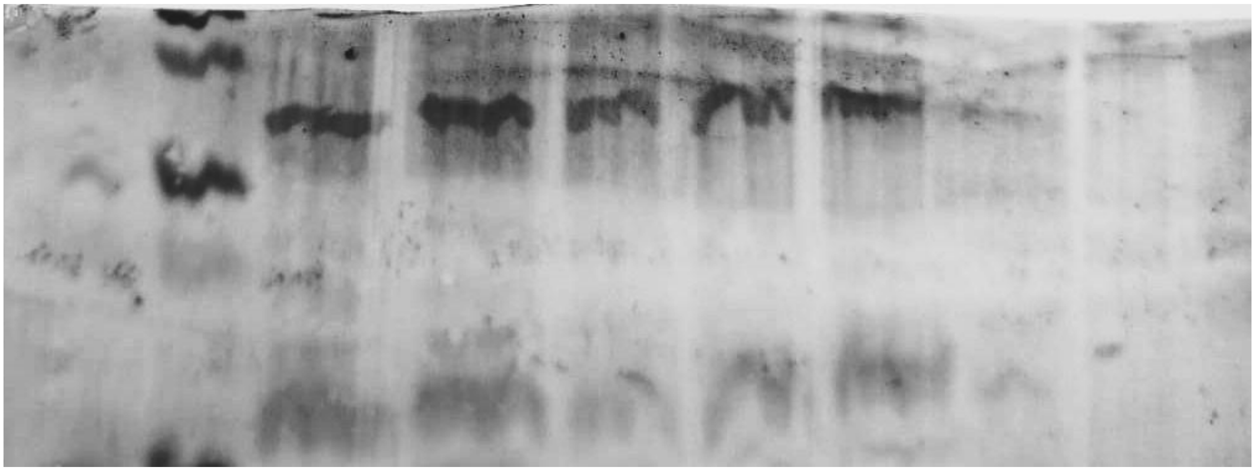

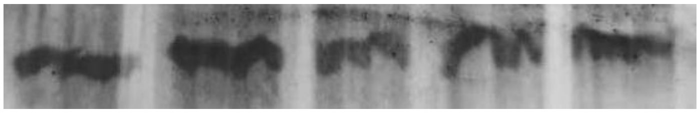

42 kDa
